# Supplementary material for: An expanded toolkit for gene tagging based on MiMIC and scarless CRISPR tagging in Drosophila
Source: eLife. 2018 Aug 9;7:e38709. doi: 10.7554/eLife.38709 (PMC6095692; doi:10.7554/eLife.38709)
Supplement: Supplementary file 1. [file elife-38709-supp1.docx]

Supplementary File 1

| oligo name | sequence |
| --- | --- |
| DLK0048 | ctagcaCTCGAGaaaaaaaccttcatataaaacgcggccgac |
| DLK0049 | ctagcaTCTAGAcctgcaggtcaacggatcctac |
| DLK0022 | See MCS of pattB ywing2+ |
| DLK0023 | See MCS of pattB ywing2+ (reverse complement) |
| DLK0054 | ctgacGCGGCCGCatgattatcgcccgattaccacattgagtg |
| DLK0055 | ctgacGCGGCCGCctctattcaatcgggacagtggaaattgac |
| DLK0056 | atcgGTCGACtccacaaatcagatctctttggccataattg |
| DLK0057 | atcgGTCGACgtcaatttccactgtcccgattgaatagag |
| DLK0225 | CCGCGGTGGGAAGACttGGAGGTTCCGGTGGAA  GCGGAGGTAGCGGCggatccGTGTCCAAGGG |
| DLK300 | GAGAGTCTAGAAGAGAGGTCTCGCTTGTACAGCTCATCCATGCCCAG |
| DLK320 | ggTGGCGTCTCtGGATgattaactagtgTTCCCGAGACGGAT |
| DLK322 | ggTGGGAAGACttGGATgattaactagtgTTCCCcGTCTTCGAT |
| DLK324 | ggTGGGGTCTCtGGATgattaactagtgTTCCCGAGACCGAT |
| DLK326 | GAGATCGGTCTCGGGAACCTGCAGGTCAACGGATCCTAC |
| DLK327 | ggTGGGGTCTCtGGATctctattcaatcgggacagtggaaattgac |
| DLK338 | ATCGAAGACGGGGAACACTAGTTAATCATCCAAGTCTTCCCACCGC |
| DLK339 | ATCGGTCTCGGGAACACTAGTTAATCATCCAGAGACCCCACCGC |
| DLK340 | ATCCGTCTCGGGAACACTAGTTAATCATCCAGAGACGCCACCGC |
| DLK461 | CGGTCTCtTCCGTCTAGATTCTcGAGACCGAT |
| DLK462 | ATCGGTCTCgAGAATCTAGACGGAaGAGACCGAGCT |
| DLK463 | CCGTCTCtTCCGTCTAGATTCTcGAGACGGAT |
| DLK464 | ATCCGTCTCgAGAATCTAGACGGAaGAGACGGAGCT |
| DLK465 | CGAAGACttTCCGTCTAGATTCTccGTCTTCGAT |
| DLK466 | ATCGAAGACggAGAATCTAGACGGAaaGTCTTCGAGCT |
| DLK554 | ggcgGATCCGGAGGTAGCGGTGGAAGCGGAGGTTCTcGAGACCtctct |
| DLK555 | ctagagagaGGTCTCgAGAACCTCCGCTTCCACCGCTACCTCCGGATC |
| DLK823 | gcgcGGATCCggcaatgattcccgcttgc |
| DLK824 | acacacGCGGCCGCcagctggttcgatagctttcg |
| DLK966 | ctctctctcgaggcgatttaacagtccgtcaag |
| DLK969 | acacaccctagggttggatcacaagttttcattgttgc |
| MiMIC_5’_for | gctaccttaatctcaagaagagcaaaacaaaagc |
| MiMIC_3’_rev | cgcggcgtaatgtgatttactatcatac |
| GFP_DH_for | gcaccacgccggtgaacag |
| T2A_GAL4_DH_rev | ctgtagcggcactcccagttgttc |

sgRNAs used in study

| Nmnat ywing2+ KI upstream | AAGCGAAAGCAATGTTCGTG |
| --- | --- |
| Nmnat ywing2+ KI downstream | CACGTTCGTTACCAAGTTGA |
| Stub1 ywing2+ KI upstream | ATTCTGTCGGCTCTTAAATT |
| Stub1 ywing2+ KI downstream | CGATATCGATAGAGAGTTAG |
| Ubqn ywing2+ KI upstream | CCGCTATCGATTCATCGAT |
| Ubqn ywing2+ KI downstream | ATAGTAGAGGGTAAAAGACG |
| Itp-r83A ywing2+ KI upstream | AGGTCCAACTAATTCACTAA |
| Itp-r83A ywing2+ KI downstream | GGTGGAGTTCTACGCTCGAT |
| CG18769 ywing2+ KI upstream | TTTGGGGAAGTGAAGGGAGT |
| CG18769 ywing2+ KI downstream | GCACACACAATATTTGCCTT |
| CG13390 ywing2+ KI upstream | AGCCCCAGGAATCCAGTTAG |
| CG13390 ywing2+ KI downstream | TTGCTGAAGTCTTGTGACCA |
| CG11679 ywing2+ KI upstream | AATGGTATAGATCTACGTTG |
| CG11679 ywing2+ KI downstream | TGGCTATATAATATAGTAGC |
| Med27 ywing2+ KI upstream | TGATGAGACGAAATTACTGT |
| Med27 ywing2+ KI downstream | TATGTACTTTGAATGCATGC |
| rho ywing2+ KI upstream | TCCAAATTCCGAGCGGTCTc |
| rho ywing2+ KI downstream | GTTTTCTGCGTCTGACTCGC |
| amx ywing2+ KI upstream | GAAGATCTTGCTATTCCTAA |
| amx ywing2+ KI downstream | TCCATTTAAGTTGTGACCAT |
| Nmnat ywing2+ replacement upstream | TCGCAAGCGAAAGCAATGTT |
| Nmnat ywing2+ replacement downstream | CAAAAGCATGGGCAGCCGTC |
| Stub1 ywing2+ replacement upstream | ATTGATTCTGTCGGCTCTTA |
| Stub1 ywing2+ replacement downstream | ATTGTTTTCACACTACCGCT |
| CG11679 ywing2+ replacement upstream | cacagcgttggccatccttt |
| CG11679 ywing2+ replacement downstream | AAAGGCAATCGGAGGCCGGC |
| Med27 ywing2+ replacement upstream | CGATTGAATAGAGATCCtgt |
| Med27 ywing2+ replacement downstream | GTTGACCTGCAGGTTCCtgc |
| amx ywing2+ replacement upstream | TTGAGAAGATCTTGCTATTC |
| amx ywing2+ replacement downstream | CTACGACTAAAGCGGCCAAT |

Sequence of Double Header constructs (Reverse orientated elements are marked with the same color but with underline)

GGCCAGACCCACGTAGTCCAGCGGCAGATCGGCGGCGGAGAAGTTAAGCGTCTCCAGGATGACCTTGCCCGAACTGGGGCACGTGGTGTTCGACGATGTGCAGCTAATTTCGCCCGGCTCCACGTCCGCCCATTGGTTAATCAGCAGACCCTCGTTGGCGTAACGGAACCATGAGAGGTACGACAACCATTTGAGGTATACTGGCACCGAGCCCGAGTTCAAGAAGAAGGCGTTTTTCCATAGGCTCCGCCCCCCTGACGAGCATCACAAAAATCGACGCTCAAGTCAGAGGTGGCGAAACCCGACAGGACTATAAAGATACCAGGCGTTTCCCCCTGGAAGCTCCCTCGTGCGCTCTCCTGTTCCGACCCTGCCGCTTACCGGATACCTGTCCGCCTTTCTCCCTTCGGGAAGCGTGGCGCTTTCTCAATGCTCACGCTGTAGGTATCTCAGTTCGGTGTAGGTCGTTCGCTCCAAGCTGGGCTGTGTGCACGAACCCCCCGTTCAGCCCGACCGCTGCGCCTTATCCGGTAACTATCGTCTTGAGTCCAACCCGGTAAGACACGACTTATCGCCACTGGCAGCAGCCACTGGTAACAGGATTAGCAGAGCGAGGTATGTAGGCGGTGCTACAGAGTTCTTGAAGTGGTGGCCTAACTACGGCTACACTAGAAGGACAGTATTTGGTATCTGCGCTCTGCTGAAGCCAGTTACCTTCGGAAAAAGAGTTGGTAGCTCTTGATCCGGCAAACAAACCACCGCTGGTAGCGGTGGTTTTTTTGTTTGCAAGCAGCAGATTACGCGCAGAAAAAAAGGATCTCAAGAAGATCCTTTGATCTTTTCTACGGGGTCTGACGCTCAGTGGAACGAAAACTCACGTTAAGGGATTTTGGTCATGAGATTATCAAAAAGGATCTTCACCTAGATCCTTTTAAATTAAAAATGAAGTTTTAAATCAATCTAAAGTATATATGAGTAAACTTGGTCTGACAGTTACCAATGCTTAATCAGTGAGGCACCTATCTCAGCGATCTGTCTATTTCGTTCATCCATAGTTGCCTGACTCCCCGTCGTGTAGATAACTACGATACGGGAGGGCTTACCATCTGGCCCCAGTGCTGCAATGATACCGCGAGACCCACGCTCACCGGCTCCAGATTTATCAGCAATAAACCAGCCAGCCGGAAGGGCCGAGCGCAGAAGTGGTCCTGCAACTTTATCCGCCTCCATCCAGTCTATTAATTGTTGCCGGGAAGCTAGAGTAAGTAGTTCGCCAGTTAATAGTTTGCGCAACGTTGTTGCCATTGCTACAGGCATCGTGGTGTCACGCTCGTCGTTTGGTATGGCTTCATTCAGCTCCGGTTCCCAACGATCAAGGCGAGTTACATGATCCCCCATGTTGTGCAAAAAAGCGGTTAGCTCCTTCGGTCCTCCGATCGTTGTCAGAAGTAAGTTGGCCGCAGTGTTATCACTCATGGTTATGGCAGCACTGCATAATTCTCTTACTGTCATGCCATCCGTAAGATGCTTTTCTGTGACTGGTGAGTACTCAACCAAGTCATTCTGAGAATAGTGTATGCGGCGACCGAGTTGCTCTTGCCCGGCGTCAACACGGGATAATACCGCGCCACATAGCAGAACTTTAAAAGTGCTCATCATTGGAAAACGTTCTTCGGGGCGAAAACTCTCAAGGATCTTACCGCTGTTGAGATCCAGTTCGATGTAACCCACTCGTGCACCCAACTGATCTTCAGCATCTTTTACTTTCACCAGCGTTTCTGGGTGAGCAAAAACAGGAAGGCAAAATGCCGCAAAAAAGGGAATAAGGGCGACACGGAAATGTTGAATACTCATACTCTTCCTTTTTCAATATTATTGAAGCATTTATCAGGGTTATTGTCTCATGAGCGGATACATATTTGAATGTATTTAGAAAAATAAACAAATAGGGGTTCCGCGCACATTTCCCCGAAAAGTGCCACCTGACGTCTAAGAAACCATTATTATCATGACATTAACCTATAAAAATAGGCGTATCACGAGGCCCTTTCGTCTCGCGCGTTTCGGTGATGACGGTGAAAACCTCTGACACATGCAGCTCCCGGAGACGGTCACAGCTTGTCTGTAAGCGGATGCCGGGAGCAGACAAGCCCGTCAGGGCGCGTCAGCGGGTGTTGGCGGGTGTCGGGGCTGGCTTAACTATGCGGCATCAGAGCAGATTGTACTGAGAGTGCACCATATGCGGTGTGAAATACCGCACCGAATCGCGCGGAACTAACGACAGTCGCTCCAAGGTCGTCGAACAAAAGGTGAATGTGTTGCGGAGAGCGGGTGGGAGACAGCGAAAGAGCAACTACGAAACGTGGTGTGGTGGAGGTGAATTATGAAGAGGGCGCGCGATTTGAAAAGTATGTATATAAAAAATATATCCCGGTGTTTTATGTAGCGATAAACGAGTTTTTGATGTAAGGTATGCAGGTGTGTAAGTCTTTTGGTTAGAAGACAAATCCAAAGTCTACTTGTGGGGATGTTCGAAGGGGAAATACTTGTATTCTATAGGTCATATCTTGTTTTTATTGGCACAAATATAATTACATTAGCTTTTTGAGGGGGCAATAAACAGTAAACACGATGGTAATAATGGTAAAAAAAAAAACAAGCAGTTATTTCGGATATATGTCGGCTACTCCTTGCGTCGGGCCCGAAGTCTTAGAGCCAGATATGCGAGCACCCGGAAGCTCACGATGAGAATGGCCAGACCATGATGAAATAACATAAGGTGGTCCCGTCGGCAAGAGACATCCACTTAACGTATGCTTGCAATAAGTGCGAGTGAAAGGAATAGTATTCTGAGTGTCGTATTGAGTCTGAGTGAGACAGCGATATGATTGTTGATTAACCCTTAGCATGTCCGTGGGGTTTGAATTAACTCATAATATTAATTAGACGAAATTATTTTTAAAGTTTTATTTTTAATAATTTGCGAGTACGCAAAGCTTGGCTGCATCCAACGCGCAATTAACCCTCACTAAAGGGAACAAAAGCTGGGTACCGGGCCCCCCCTCGAGATAACTTCGTATAATGTATGCTATACGAAGTTATCAATTGGCTTATTGGGATTTCAATTTAAGATGCTGCTAAATAAAGTTAGTCACTTACGGACTGGGGAGGCACCCCGTCATCACTCGCCAGAAATGCAATTGATGTAGGTCACGGTCTCGAAGCCGCGGTGCGGGTGCCAGGGCGTGCCCTTGGGCTCCCCGGGCGCGTACTCCACCTCACCCATCTGGTCCATCATGATGGTCGACTCTAGAAGTCGATCCAACATGGCGACTTGTCCCATCCCCGGCATGTTTAAATATACTAATTATTCTTGAACTAATTTTAATCAACCGATTTATCTCTCTTCCGCAGGTGGGAGGTTCCGGTGGAAGCGGAGGTAGCGGCGGATCCGAGGGCCGCGGCAGCCTGCTGACCTGCGGCGATGTGGAGGAGAACCCCGGGCCCATGAAGCTGCTGAGCAGCATCGAGCAGGCCTGCGATATCTGCCGCCTGAAGAAGCTGAAGTGCAGCAAGGAGAAGCCCAAGTGCGCCAAGTGCCTGAAGAACAACTGGGAGTGCCGCTACAGCCCCAAGACCAAGCGCAGCCCCCTGACCCGCGCCCACCTGACCGAGGTGGAGAGCCGCCTGGAGCGCCTGGAGCAGCTGTTCCTGCTGATCTTCCCCCGCGAGGATCTGGATATGATCCTGAAGATGGATAGCCTGCAGGATATCAAGGCCCTGCTGACCGGCCTGTTCGTGCAGGATAACGTGAACAAGGATGCCGTGACCGATCGCCTGGCCAGCGTGGAGACCGATATGCCCCTGACCCTGCGCCAGCACCGCATCAGCGCCACCAGCAGCAGCGAGGAGAGCAGCAACAAGGGCCAGCGCCAGCTGACCGTGAGCATCGATAGCGCCGCCCACCACGATAACAGCACCATCCCCCTGGATTTCATGCCCCGCGATGCCCTGCACGGCTTCGATTGGAGCGAGGAGGATGATATGAGCGATGGCCTGCCCTTCCTGAAGACCGATCCCAACAACAACGGCTTCTTCGGCGATGGCAGCCTGCTGTGCATCCTGCGCAGCATCGGCTTCAAGCCCGAGAACTACACCAACAGCAACGTGAACCGCCTGCCCACCATGATCACCGATCGCTACACCCTGGCCAGCCGCAGCACCACCAGCCGCCTGCTGCAGAGCTACCTGAACAACTTCCACCCCTACTGCCCCATCGTGCACAGCCCCACCCTGATGATGCTGTACAACAACCAGATCGAGATCGCCAGCAAGGATCAGTGGCAGATCCTGTTCAACTGCATCCTGGCCATCGGCGCCTGGTGCATCGAGGGCGAGAGCACCGATATCGATGTGTTCTACTACCAGAACGCCAAGAGCCACCTGACCAGCAAGGTGTTCGAGAGCGGCAGCATCATCCTGGTGACCGCCCTGCACCTGCTGAGCCGCTACACCCAGTGGCGCCAGAAGACCAACACCAGCTACAACTTCCACAGCTTCAGCATCCGCATGGCCATCAGCCTGGGCCTGAACCGCGATCTGCCCAGCAGCTTCAGCGATAGCAGCATCCTGGAGCAGCGCCGCCGCATCTGGTGGAGCGTGTACAGCTGGGAGATCCAGCTGAGCCTGCTGTACGGCCGCAGCATCCAGCTGAGCCAGAACACCATCAGCTTCCCCAGCAGCGTGGATGATGTGCAGCGCACCACCACCGGCCCCACCATCTACCACGGCATCATCGAGACCGCCCGCCTGCTGCAGGTGTTCACCAAGATCTACGAGCTGGATAAGACCGTGACCGCCGAGAAGAGCCCCATCTGCGCCAAGAAGTGCCTGATGATCTGCAACGAGATCGAGGAGGTGAGCCGCCAGGCCCCCAAGTTCCTGCAGATGGATATCAGCACCACCGCCCTGACCAACCTGCTGAAGGAGCACCCCTGGCTGAGCTTCACCCGCTTCGAGCTGAAGTGGAAGCAGCTGAGCCTGATCATCTACGTGCTGCGCGATTTCTTCACCAACTTCACCCAGAAGAAGAGCCAGCTGGAGCAGGATCAGAACGATCACCAGAGCTACGAGGTGAAGCGCTGCAGCATCATGCTGAGCGATGCCGCCCAGCGCACCGTGATGAGCGTGAGCAGCTACATGGATAACCACAACGTGACCCCCTACTTCGCCTGGAACTGCAGCTACTACCTGTTCAACGCCGTGCTGGTGCCCATCAAGACCCTGCTGAGCAACAGCAAGAGCAACGCCGAGAACAACGAGACCGCCCAGCTGCTGCAGCAGATCAACACCGTGCTGATGCTGCTGAAGAAGCTGGCCACCTTCAAGATCCAGACCTGCGAGAAGTACATCCAGGTGCTGGAGGAGGTGTGCGCCCCCTTCCTGCTGAGCCAGTGCGCCATCCCCCTGCCCCACATCAGCTACAACAACAGCAACGGCAGCGCCATCAAGAACATCGTGGGCAGCGCCACCATCGCCCAGTACCCCACCCTGCCCGAGGAGAACGTGAACAACATCAGCGTGAAGTACGTGAGCCCCGGCAGCGTGGGACCCAGCCCCGTGCCCCTGAAGAGCGGCGCCAGCTTCAGCGATCTGGTGAAGCTGCTGAGCAACCGCCCCCCCAGCCGCAACAGCCCCGTGACCATCCCCCGCAGCACCCCCAGCCACCGCAGCGTGACCCCCTTCCTGGGCCAGCAGCAGCAGCTGCAGAGCCTGGTGCCCCTGACCCCCAGCGCCCTGTTCGGCGGCGCCAACTTCAACCAGAGCGGCAACATCGCCGATAGCAGCCTGAGCTTCACCTTCACCAACAGCAGCAACGGCCCCAACCTGATCACCACCCAGACCAACAGCCAGGCCCTGAGCCAGCCCATCGCCAGCAGCAACGTGCACGATAACTTCATGAACAACGAGATCACCGCCAGCAAGATCGATGATGGCAACAACAGCAAGCCCCTGAGCCCCGGCTGGACCGATCAGACCGCCTACAACGCCTTCGGCATCACCACCGGCATGTTCAACACCACCACCATGGATGATGTGTACAACTACCTGTTCGATGATGAGGATACCCCCCCCAACCCCAAGAAGGAGTAAAATGAATCGTAGATACTGAAAAACCCCGCAAGTTCACTTCAACTGTGCATCGTGCACCATCTCAATTTCTTTCATTTATACATCGTTTTGCCTTCTTTTATGTAACTATACTCCTCTAAGTTTCAATCTTGGCCATGTAACCTCTGATCTATAGAATTTTTTAAATGACTAGAATTAATGCCCATCTTTTTTTTGGACCTAAATTCTTCATGAAAATATATTACGAGGGCTTATTCAGAAGCTTATCGATACCGTCGACTAAAGCCAAATAGAAATTATTCAGTTCTGGCTTAAGTTTTTAAAAGTGATATTATTTATTTGGTTGTAACCAACCAAAAGAATGTAAATAACTAATACATAATTATGTTAGTTTTAAGTTAGCAACAAATTGATTTTAGCTATATTAGCTACTTGGTTAATAAATAGAATATATTTATTTAAAGATAATTCGTTTTTATTGTCAGGGAGTGAGTTTGCTTAAAAACTCGTTTGGATCCGACGTCTAATCCTAGGAGAAGTTCAAATGGGCTTTCATATTTACTTTTTTCTCTGAGTGGGTAAGGTCATTGTGGGGTCTTAAGGGCTAAAGGGTAGTGATGATGAAGGCATTTGATGCCATTGTTCAATAACTTACCTCGCCGGAACCTCCGCTTCCACCGCTACCTCCGGATCCGCCCTTGTCGTCATCGTCCTTGTAATCAATATCGTGGTCCTTGTAGTCGCCGTCGTGATCCTTGTAATCGCTCTGGAAGTACAGGTTTTCCTTCTCGAACTGGGGGTGGGACCAGTTGCCGCTTCCCGGTTCCATGCAGCATCCTGGGCAGCAGTTCAGGAAGCTGCCGCTGCTCTCCTTGTACAGCTCATCCATGCCCAGGGTGATGCCGGCGGCGGTCACGAACTCCAGCAGCACCATGTGATCGCGCTTCTCGTTGGGGTCCTTGGACAGCACGCTCTGGGTGCTCAGGTAGTGGTTATCGGGCAGCAGCACTGGGCCGTCGCCGATGGGGGTGTTCTGCTGGTAGTGATCGGCCAGCTGCACGGAGCCATCCTCCACATTGTGGCGGATCTTGAAGTTGGCCTTGATGCCGTTCTTCTGCTTATCGGCGGTGATGTACACGTTGTGGCTGTTGAAGTTGTACTCCAGCTTGTGGCCCAGGATGTTGCCATCCTCCTTGAAATCGATGCCCTTCAGCTCGATGCGGTTCACCAGGGTATCGCCCTCGAACTTCACCTCGGCGCGGGTCTTGTAGGTGCCGTCATCCTTGAAGCTGATGGTGCGCTCCTGCACGTAGCCCTCGGGCATGGCGCTCTTGAAGAAATCGTGCTGCTTCATGTGATCGGGGTAGCGGCTGAAGCACTGCACGCCGTAGGTCAGGGTGGTCACCAGGGTGGGCCAGGGCACGGGCAGCTTGCCGGTGGTGCAGATGAACTTCAGGGTCAGCTTGCCGTTGGTGGCGTCGCCCTCGCCCTCGCCGCGCACGCTGAACTTGTGGCCGTTCACGTCGCCATCCAGCTCCACCAGGATGGGCACCACGCCGGTGAACAGCTCCTCGCCCTTGGACACGGATCCGCCGCTACCTCCGCTTCCACCGGAACCTCCCACCTGCGGAAGAGAGATAAATCGGTTGATTAAAATTAGTTCAAGAATAATTAGTATATTTAAACATGCCGGGGATGGGACAAGTCGCCATGTTGGATCGACTTCCGTACGCTTGTCGACCATCATGATGGACCAGATGGGTGAGGTGGAGTACGCGCCCGGGGAGCCCAAGGGCACGCCCTGGCACCCGCACCGCGGCTTCGAGACCGTGACCTACATCATACAATAAATATTCACACCCCTAAAAAACGAAGCTGCATTTGCAAATTGCATACAATTATGCCAGAGTAAAAATTATAATTGTTATTATTTAAATTTGAATAACTTCGTATAATGTATGCTATACGAAGTTATAGATCCACTAGAAGGCCTAATTCTAGTATGTATGTAAGTTAATAAAACCCATTTTTGCGGAAAGTAGATAAAAAAAACATTTTTTTTTTTTACTGCACTGGATATCATTGAACTTATCTGATCAGTTTTAAATTTACTTCGATCCAAGGGTATTTGATGTACCAGGTTCTTTCGATTACCTCTCACTCAAAATGACATTCCACTCAAAGTCAGCGCTGTTTGCCTCCTTCTCTGTCCACAGAAATATCGCCGTCTCTTTCGCCGCTGCGTCCGCTATCTCTTTCGCCACCGTTTGTAGCGTTACCTAGCGTCAATGTCCGCCTTCAGTTGCACTTTGTCAGCGGTTTCGTGACGAAGCTCCAAGCGGTTTACGCCATCAATTAAACACAAAGTGCTGTGCCAAAACTCCTCTCGCTTCTTATTTTTGTTTGTTTTTTGAGTGATTGGGGTGGTGATTGGTTTTGGGTGGGTAAGCAGGGGAAAGTGTGAAAAATCCCGGCAATGGGCCAAGAGGATCAGGAGCTATTAATTCGCGGAGGCAGCAAACACCCATCTGCCGAGCATCTGAACAATGTGAGTAGTACATGTGCATACATCTTAAGTTCACTTGATCTATAGGAACTGCGATTGCAACATCAAATTGTCTGCGGCGTGAGAACTGCGACCCACAAAAATCCCAAACCGCAATCGCACAAACAAATAGTGACACGAAACAGATTATTCTGGTAGCTGTGCTCGCTATATAAGACAATTTTTAAGATCATATCATGATCAAGACATCTAAAGGCATTCATTTTCGACTACATTCTTTTTTACAAAAAATATAACAACCAGATATTTTAAGCTGATCCTAGATGCACAAAAAATAAATAAAAGTATAAACCTACTTCGTAGGATACTTCGTTTTGTTCGGGGTTAGATGAGCATAACGCTTGTAGTTGATATTTGAGATCCCCTATCATTGCAGGGTGACAGCGGAGCGGCTTCGCAGAGCTGCATTAACCAGGGCTTCGGGCAGGCCAAAAACTACGGCACGCTCCTGCCACCCAGTCCGCCGGAGGACTCCGGTTCAGGGAGCGGCCAACTAGCCGAGAACCTCACCTATGCCTGGCACAATATGGACATCTTTGGGGCGGTCAATCAGCCGGGCTCCGGATGGCGGCAGCTGGTCAACCGGACACGCGGACTATTCTGCAACGAGCGACACATACCGGCGCCCAGGAAACATTTGCTCAAGAACGGTGAGTTTCTATTCGCAGTCGGCTGATCTGTGTGAAATCTTAATAAAGGGTCCAATTACCAATTTGAAACTCAGTTTGCGGCGTGGCCTATCCGGGCGAACTTTTGGCCGTGATGGGCAGTTCCGGTGCCGGAAAGACGACCCTGCTGAATGCCCTTGCCTTTCGATCGCCGCAGGGCATCCAAGTATCGCCATCCGGGATGCGACTGCTCAATGGCCAACCTGTGGACGCCAAGGAGATGCAGGCCAGGTGCGCCTATGTCCAGCAGGATGACCTCTTTATCGGCTCCCTAACGGCCAGGGAACACCTGATTTTCCAGGCCATGGTGCGGATGCCACGACATCTGACCTATCGGCAGCGAGTGGCCCGCGTGGATCAGGTGATCCAGGAGCTTTCGCTCAGCAAATGTCAGCACACGATCATCGGTGTGCCCGGCAGGGTGAAAGGTCTGTCCGGCGGAGAAAGGAAGCGTCTGGCATTCGCCTCCGAGGCACTAACCGATCCGCCGCTTCTGATCTGCGATGAGCCCACCTCCGGACTGGACTCATTTACCGCCCACAGCGTCGTCCAGGTGCTGAAGAAGCTGTCGCAGAAGGGCAAGACCGTCATCCTGACCATTCATCAGCCGTCTTCCGAGCTGTTTGAGCTCTTTGACAAGATCCTTCTGATGGCCGAGGGCAGGGTAGCTTTCTTGGGCACTCCCAGCGAAGCCGTCGACTTCTTTTCCTAGTGAGTTCGATGTGTTTATTAAGGGTATCTAGCATTACATTACATCTCAACTCCTATCCAGCGTGGGTGCCCAGTGTCCTACCAACTACAATCCGGCGGACTTTTACGTACAGGTGTTGGCCGTTGTGCCCGGACGGGAGATCGAGTCCCGTGATCGGATCGCCAAGATATGCGACAATTTTGCTATTAGCAAAGTAGCCCGGGATATGGAGCAGTTGTTGGCCACCAAAAATTTGGAGAAGCCACTGGAGCAGCCGGAGAATGGGTACACCTACAAGGCCACCTGGTTCATGCAGTTCCGGGCGGTCCTGTGGCGATCCTGGCTGTCGGTGCTCAAGGAACCACTCCTCGTAAAAGTGCGACTTATTCAGACAACGGTGAGTGGTTCCAGTGGAAACAAATGATATAACGCTTACAATTCTTGGAAACAAATTCGCTAGATTTTAGTTAGAATTGCCTGATTCCACACCCTTCTTAGTTTTTTTCAATGAGATGTATAGTTTATAGTTTTGCAGAAAATAAATAAATTTCATTTAACTCGCGAACATGTTGAAGATATGAATATTAATGAGATGCGAGTAACATTTTAATTTGCAGATGGTTGCCATCTTGATTGGCCTCATCTTTTTGGGCCAACAACTCACGCAAGTGGGCGTGATGAATATCAACGGAGCCATCTTCCTCTTCCTGACCAACATGACCTTTCAAAACGTCTTTGCCACGATAAATGTAAGTCTTGTTTAGAATACATTTGCATATTAATAATTTACTAACTTTCTAATGAATCGATTCGATTTAGGTGTTCACCTCAGAGCTGCCAGTTTTTATGAGGGAGGCCCGAAGTCGACTTTATCGCTGTGACACATACTTTCTGGGCAAAACGATTGCCGAATTACCGCTTTTTCTCACAGTGCCACTGGTCTTCACGGCGATTGCCTATCCGATGATCGGACTGCGGGCCGGAGTGCTGCACTTCTTCAACTGCCTGGCGCTGGTCACTCTGGTGGCCAATGTGTCAACGTCCTTCGGATATCTAATATCCTGCGCCAGCTCCTCGACCTCGATGGCGCTGTCTGTGGGTCCGCCGGTTATCATACCATTCCTGCTCTTTGGCGGCTTCTTCTTGAACTCGGGCTCGGTGCCAGTATACCTCAAATGGTTGTCGTACCTCTCATGGTTCCGTTACGCCAACGAGGGTCTGCTGATTAACCAATGGGCGGACGTGGAGCCGGGCGAAATTAGCTGCACATCGTCGAACACCACGTGCCCCAGTTCGGGCAAGGTCATCCTGGAGACGCTTAACTTCTCCGCCGCCGATCTGCCGCTGGACTACGTGGGTCTGGCCATTCTCATCGTGAGCTTCCGGGTGCTCGCATATCTGGCTCTAAGACTTCGGGCCCGACGCAAGGAGTAGCCGACATATATCCGAAATAACTGCTTGTTTTTTTTTTTACCATTATTACCATCGTGTTTACTGTTTATTGCCCCCTCAAAAAGCTAATGTAATTATATTTGTGCCAATAAAAACAAGATATGACCTATAGAATACAAGTATTTCCCCTTCGAACATCCCCACAAGTAGACTTTGGATTTGTCTTCTAACCAAAAGACTTACACACCTGCATACCTTACATCAAAAACTCGTTTATCGCTACATAAAACACCGGGATATATTTTTTATATACATACTTTTCAAATCGCGCGCCCTCTTCATAATTCACCTCCACCACACCACGTTTCGTAGTTGCTCTTTCGCTGTCTCCCACCCGCTCTCCGCAACACATTCACCTTTTGTTCGACGACCTTGGAGCGACTGTCGTTAGTTCCGCGCGATTCGGTTCGCTCAAATGGTTCCGAGTGGTTCATTTCGTCTCAATAGAAATTAGTAATAAATATTTGTATGTACAATTTATTTGCTCCAATATATTTGTATATATTTCCCTCACAGCTATATTTATTCTAATTTAATATTATGACTTTTTAAGGTAATTTTTTGTGACCTGTTCGGAGTGATTAGCGTTACAATTTGAACTGAAAGTGACATCCAGTGTTTGTTCCTTGTGTAGATGCATCTCAAAAAAATGGTGGGCATAATAGTGTTGTTTATATATATCAAAAATAACAACTATAATAATAAGAATACATTTAATTTAGAAAATGCTTGGATTTCACTGGAACTAGAATTAATTCGGCTGCTGCTCTAAACGACGCATTTCGTACTCCAAAGTACGAATTTTTTCCCTCAAGCTCTTATTTTCATTAAACAATGAACAGGACCTAACGCACAGTCACGTTATTGTTTACATAAATGATTTTTTTTACTATTCAAACTTACTCTGTTTGTGTACTCCCACTGGTATAGCCTTCTTTTATCTTTTCTGGTTCAGGCTCTATCACTTTACTAGGTACGGCATCTGCGTTGAGTCGCCTCCTTTTAAATGTCTGACCTTTTGCAGGTGCAGCCTTCCACTGCGAATCATTAAAGTGGGTATCACAAATTTGGGAGTTTTCACCAAGGCTGCACCCAAGGCTCTGCTCCCACAATTTTCTCTTAATAGCACACTTCGGCACGTGAATTAATTTTACTCCAGTCACAGCTTTGCAGCAAAATTTGCAATATTTCATTTTTTTTTATTCCACGTAAGGGTTAATGTTTTCAAAAAAAAATTCGTCCGCACACAACCTTTCCTCTCAACAAGCAAACGTGCACTGAATTTAAGTGTATACTTCGGTAAGCTTCGGCTATCGACGGGACCACCTTATGTTATTTCATCATG

NNNN Origin of replication

NNNN P-Element inverted repeats

NNNN LoxP

NNNN attB

NNNN MHC splice acceptor

NNNN (GSS)_4_ linker*

NNNN Amp resistance

NNNN T2A-GAL4_polyA

NNNN Splice donor

NNNN EGFP-FIAsH tag-StrepII tag-TEV protease cleavage site-3XFlag

NNNN white

*Linker for p1 GTGGCGGAGGTTCCGGTGGAAGCGGAGGTAGCGGCGGATCC

for p2 GTCGGGAGGTTCCGGTGGAAGCGGAGGTAGCGGCGGATCC

(sense orientations)

for p1 SA-GGATCCGCCGCTACCTCCGCTTCCACCGGAACCTCCGCCAC

/…/ CTCCGGAACCTCCGCTTCCACCGCTACCTCCGGATCC-SD

for p2 SA-GGATCCGCCGCTACCTCCGCTTCCACCGGAACCTCCCGAC

/…/ CTGCCGGAACCTCCGCTTCCACCGCTACCTCCGGATCC-SD

(antisense orientation (around EGFP-FlAsH tag-StrepII tag-TEV-3XFlag cassette))

Sequence of y^wing2+^ donor template vector- BbsI version

CTAAATTGTAAGCGTTAATATTTTGTTAAAATTCGCGTTAAATTTTTGTTAAATCAGCTCATTTTTTAACCAATAGGCCGAAATCGGCAAAATCCCTTATAAATCAAAAGAATAGACCGAGATAGGGTTGAGTGTTGTTCCAGTTTGGAACAAGAGTCCACTATTAAAGAACGTGGACTCCAACGTCAAAGGGCGAAAAACCGTCTATCAGGGCGATGGCCCACTACGTGAACCATCACCCTAATCAAGTTTTTTGGGGTCGAGGTGCCGTAAAGCACTAAATCGGAACCCTAAAGGGAGCCCCCGATTTAGAGCTTGACGGGGAAAGCCGGCGAACGTGGCGAGAAAGGAAGGGAAGAAAGCGAAAGGAGCGGGCGCTAGGGCGCTGGCAAGTGTAGCGGTCACGCTGCGCGTAACCACCACACCCGCCGCGCTTAATGCGCCGCTACAGGGCGCGTCCCATTCGCCATTCAGGCTGCGCAACTGTTGGGAAGGGCGATCGGTGCGGGCCTCTTCGCTATTACGCCAGCTGGCGAAAGGGGGATGTGCTGCAAGGCGATTAAGTTGGGTAACGCCAGGGTTTTCCCAGTCACGACGTTGTAAAACGACGGCCAGTGAGCGCGCGTAATACGACTCACTATAGGGCGAATTGGAGCTCCACCGCggTGGGAAGACttGGATCTCTATTCAATCGGGACAGTGGAAATTGACTATTTTATTTATATTAATGAACTTATTTTTAATTTGGCTTAAGTTACTAAGGGGTACTAATAGTTTGAGCGCAGTGCATGTCATGGGGACATGTGCAATTGTGTGTAAGCGGGAAGTGATCGCGGCCTTCCGAATTTGGCCATGCCAAATAATCCCAGCTCGAAAGGAGGGGACCCGGCGGTCAGGGCCATGGACATTGAACTTGAAAAAAAAAAACACAAAAATATATAACACAAAACGGAAAATGCTGTGTACCGCTTATGTTAGAGAAGTTGAGCAACGGGTTTTTCGTTTTGCAGTCACGATGGATTTCCAAATTAGTGTAGGAGGGGGGAGGGGAGGGAGGGAGATAATGTCCAGGCTGCCATAAGTGGGGAATAAGGAAAATAAAACATGAAACACGGGTCGGGCAATGTCATGCGGTATTCGGCTTTGCTTTCCGCCCAAGTTGAAGTGATCCTGTGTGTAAATAATGTCGAATGTTGCCGGTCGGTTGCATAAGCGTTAGTcaattatggccaaagagatctgatttgtggaGTCGAGAAAAAAACCTTCATATAAAACGCGGCCGACATATTATGGCCACCAGTCGTTACCGCGCCACGGTCCACAGAAGAGGATTAAAAAAATATCACACAGCCGAAGGCTAGAGAAGAACCCCCTATAGCTGAACATATATAAACAAATATATTTTTTTTTATTGCCAACACACTTTGGCTTAAGTGTTAAGAGTGATTGTCAGCTTAGAGCTAAGTGCAATGTTCCAGGACAAAGGGTGGATCCTTGTGACCCTGATCACCTTGGTGACGCCGTCTTGGGCTGCTTACAAACTTCAGGAGCGATATAGTTGGAGCCAGCTGGACTTTGCTTTCCCGAATACCCGACTAAAGGACCAAGCTCTGGCTAGTGGAGATTATATTCCGCAAAATGCTCTACCTGTTGGAGTCGAACACTTTGGCAATCGGTTATTCGTCACTGTTCCCCGCTGGCGTGATGGGATTCCGGCCACTCTGACCTATATAAACATGGACCGCAGTTTGACGGGTTCACCGGAGCTAATTCCGTATCCAGATTGGCGCTCAAATACAGCTGGAGATTGCGCCAACAGTATTACCACTGCCTACCGCATTAAAGTGGATGAGTGTGGTCGGCTGTGGGTTTTGGACACTGGAACCGTGGGCATCGGCAATACCACCACTAATCCGTGCTCCTATGCGGTAAATGTCTTTGACTTGACCACGGATACGCGAATTCGGAGATACGAGCTACCTGGCGTGGACACAAATCCAAATACTTTCATAGCTAACATTGCCGTGGATATAGGCAAAAATTGCGATGATGCATATGCCTATTTTGCCGATGAATTGGGATACGGCTTGATTGCTTACTCCTGGGAACTGAACAAGTCCTGGAGATTCTCGGCACATTCGTATTTTTTCCCCGATCCATTGAGGGGCGATTTCAATGTCGCTGGTATTAACTTCCAATGGGGCGAGGAGGGTATATTTGGTATGTCCCTTTCGCCCATTCGATCGGATGGTTATCGTACCCTGTACTTTAGTCCGTTAGCAAGTCATCGACAATTTGCCGTATCCACGAGGATTTTGAGGGATGAAACCAGGACGGAAGATAGCTATCATGACTTTGTTGCCTTAGATGAACGGGGTCCAAACTCCCATACCACTTCACGTGTGATGAGCGATGATGGAATTGAGCTGTTCAATTTAATAGATCAAAATGCAGTGGGTTGCTGGCACTCATCAATGCCGTACTCACCGCAATTTCATGGCATTGTGGATCGCGATGACGTTGGCTTAGTTTTTCCGGCCGATGTGAAAATTGATGAGAACAAAAACGTTTGGGTTCTATCCGATAGGATGCCCGTTTTCTTGCTGTCTGACTTGGATTATTCAGATACTAATTTCCGAATTTACACGGCTCCCTTGGCCACTTTAATTGAGAATACTGTGTGTGATTTGAGGAATAACGCCTATGGGCCGCCAAATACCGTTTCAATACCAAAACAAGCCGTTTTGCCAATGGGTCCACCGTTATATACGAAACAATATCGTCCTGTCTTGCCACAGAAACCTCAGACCAGCTGGGCTTCCTCGCCGCCTCCTCCAAGTCGCACTTATTTGCCCGCCAATTCAGGCAATGTAGTCTCCAGTATTAGTGTCTCTACAAATTCTGTGGGTCCTGCAGGAGTGGAGGTGCCAAAGGCCTATATTTTCAACCAGCACAACGGCATAAATTACGAGACAAGTGGTCCCCATCTATTTCCCACCCATCAACCCGCCCAACCGGGTGGCCAGGATGGTGGGTTAAAAACTTATGTGAATGCCCGCCAATCTGGGTGGTGGCATCATCAGCATCAAGGTTAACATAATCCTACACACGGTACTTGGGTATATTCTCACACACTCGATTGATGTAAAGAATATTTAAAGACAACAACATAGGGCAACAGCGGTTAAAAAAACCACATGACGTATGAGCAAGTGGCAAATCAATACTTTATCTAGTTATGTTAAGCAAAAAATAACAATAAATCAACTTTTTTTTGAAGGTTAAGAGTTTACGCAATTTTCTTGAGCGGAAAAAGCGGAAAAAATGTAAGTATGCATAAATTCTAAATATATCAACAACTGTACATTTTCTGGAGTACTACTACCAGGCAAGAAAGTAGGTTGATAAAGCTATGCACAAGATCTTGTTTGGGTGCAGGGAAAGTTCAACTTAATCGCTCAATTTGAGATCGCCTGGTCGCTTGAGATTCGACTGTAATTGAAATTTTTGCTTTTGATCGGAGCCAGACTTCAGACGGGGCAAACAAAAAGACTTTGTTGGTGGTAGGGTAGGATCCGTTGACCTGCAGGTTCCccGTCTTCGATATCAAGCTTATCGATACCGTCGACCTCGAGGGGGGGCCCGGTACCCAGCTTTTGTTCCCTTTAGTGAGGGTTAATTGCGCGCTTGGCGTAATCATGGTCATAGCTGTTTCCTGTGTGAAATTGTTATCCGCTCACAATTCCACACAACATACGAGCCGGGAGCATAAAGTGTAAAGCCTGGGGTGCCTAATGAGTGAGCTAACTCACATTAATTGCGTTGCGCTCACTGCCCGCTTTCCAGTCGGGAAACCTGTCGTGCCAGCTGCATTAATGAATCGGCCAACGCGCGGGGAGAGGCGGTTTGCGTATTGGGCGCTCTTCCGCTTCCTCGCTCACTGACTCGCTGCGCTCGGTCGTTCGGCTGCGGCGAGCGGTATCAGCTCACTCAAAGGCGGTAATACGGTTATCCACAGAATCAGGGGATAACGCAGGAAAGAACATGTGAGCAAAAGGCCAGCAAAAGGCCAGGAACCGTAAAAAGGCCGCGTTGCTGGCGTTTTTCCATAGGCTCCGCCCCCCTGACGAGCATCACAAAAATCGACGCTCAAGTCAGAGGTGGCGAAACCCGACAGGACTATAAAGATACCAGGCGTTTCCCCCTGGAAGCTCCCTCGTGCGCTCTCCTGTTCCGACCCTGCCGCTTACCGGATACCTGTCCGCCTTTCTCCCTTCGGGAAGCGTGGCGCTTTCTCATAGCTCACGCTGTAGGTATCTCAGTTCGGTGTAGGTCGTTCGCTCCAAGCTGGGCTGTGTGCACGAACCCCCCGTTCAGCCCGACCGCTGCGCCTTATCCGGTAACTATCGTCTTGAGTCCAACCCGGTAAGACACGACTTATCGCCACTGGCAGCAGCCACTGGTAACAGGATTAGCAGAGCGAGGTATGTAGGCGGTGCTACAGAGTTCTTGAAGTGGTGGCCTAACTACGGCTACACTAGAAGGACAGTATTTGGTATCTGCGCTCTGCTGAAGCCAGTTACCTTCGGAAAAAGAGTTGGTAGCTCTTGATCCGGCAAACAAACCACCGCTGGTAGCGGTGGTTTTTTTGTTTGCAAGCAGCAGATTACGCGCAGAAAAAAAGGATCTCAAGAAGATCCTTTGATCTTTTCTACGGGGTCTGACGCTCAGTGGAACGAAAACTCACGTTAAGGGATTTTGGTCATGAGATTATCAAAAAGGATCTTCACCTAGATCCTTTTAAATTAAAAATGAAGTTTTAAATCAATCTAAAGTATATATGAGTAAACTTGGTCTGACAGTTACCAATGCTTAATCAGTGAGGCACCTATCTCAGCGATCTGTCTATTTCGTTCATCCATAGTTGCCTGACTCCCCGTCGTGTAGATAACTACGATACGGGAGGGCTTACCATCTGGCCCCAGTGCTGCAATGATACCGCGtGACCCACGCTCACCGGCTCCAGATTTATCAGCAATAAACCAGCCAGCCGGAAGGGCCGAGCGCAGAAGTGGTCCTGCAACTTTATCCGCCTCCATCCAGTCTATTAATTGTTGCCGGGAAGCTAGAGTAAGTAGTTCGCCAGTTAATAGTTTGCGCAACGTTGTTGCCATTGCTACAGGCATCGTGGTGTCACGCTCGTCGTTTGGTATGGCTTCATTCAGCTCCGGTTCCCAACGATCAAGGCGAGTTACATGATCCCCCATGTTGTGCAAAAAAGCGGTTAGCTCCTTCGGTCCTCCGATCGTTGTCAGAAGTAAGTTGGCCGCAGTGTTATCACTCATGGTTATGGCAGCACTGCATAATTCTCTTACTGTCATGCCATCCGTAAGATGCTTTTCTGTGACTGGTGAGTACTCAACCAAGTCATTCTGAGAATAGTGTATGCGGCGACCGAGTTGCTCTTGCCCGGCGTCAATACGGGATAATACCGCGCCACATAGCAGAACTTTAAAAGTGCTCATCATTGGAAAACGTTCTTCGGGGCGAAAACTCTCAAGGATCTTACCGCTGTTGAGATCCAGTTCGATGTAACCCACTCGTGCACCCAACTGATCTTCAGCATCTTTTACTTTCACCAGCGTTTCTGGGTGAGCAAAAACAGGAAGGCAAAATGCCGCAAAAAAGGGAATAAGGGCGACACGGAAATGTTGAATACTCATACTCTTCCTTTTTCAATATTATTGAAGCATTTATCAGGGTTATTGTCTCATGAGCGGATACATATTTGAATGTATTTAGAAAAATAAACAAATAGGGGTTCCGCGCACATTTCCCCGAAAAGTGCCAC

NNNN y^wing2+^

Sequence of the pATTBy^wing2+^ vector

CTGACGCGCCCTGTAGCGGCGCATTAAGCGCGGCGGGTGTGGTGGTTACGCGCAGCGTGACCGCTACACTTGCCAGCGCCCTAGCGCCCGCTCCTTTCGCTTTCTTCCCTTCCTTTCTCGCCACGTTCGCCGGCTTTCCCCGTCAAGCTCTAAATCGGGGGCTCCCTTTAGGGTTCCGATTTAGTGCTTTACGGCACCTCGACCCCAAAAAACTTGATTAGGGTGATGGTTCACGTAGTGGGCCATCGCCCTGATAGACGGTTTTTCGCCCTTTGACGTTGGAGTCCACGTTCTTTAATAGTGGACTCTTGTTCCAAACTGGAACAACACTCAACCCTATCTCGGTCTATTCTTTTGATTTATAAGGGATTTTGCCGATTTCGGCCTATTGGTTAAAAAATGAGCTGATTTAACAAAAATTTAACGCGAATTTTAACAAAATATTAACGCTTACAATTTCCATTCGCCATTCAGGCTGCGCAACTGTTGGGAAGGGCGATCGGTGCGGGCCTCTTCGCTATTACGCCAGCTGGCGAAAGGGGGATGTGCTGCAAGGCGATTAAGTTGGGTAACGCCAGGGTTTTCCCAGTCACGACGTTGTAAAACGACGGCCAGTGAATTGTAATACGACTCACTATAGGGCGAATTGGGTACGTACCGGCCGCCTCTATTCAATCGGGACAGTGGAAATTGACTATTTTATTTATATTAATGAACTTATTTTTAATTTGGCTTAAGTTACTAAGGGGTACTAATAGTTTGAGCGCAGTGCATGTCATGGGGACATGTGCAATTGTGTGTAAGCGGGAAGTGATCGCGGCCTTCCGAATTTGGCCATGCCAAATAATCCCAGCTCGAAAGGAGGGGACCCGGCGGTCAGGGCCATGGACATTGAACTTGAAAAAAAAAAACACAAAAATATATAACACAAAACGGAAAATGCTGTGTACCGCTTATGTTAGAGAAGTTGAGCAACGGGTTTTTCGTTTTGCAGTCACGATGGATTTCCAAATTAGTGTAGGAGGGGGGAGGGGAGGGAGGGAGATAATGTCCAGGCTGCCATAAGTGGGGAATAAGGAAAATAAAACATGAAACACGGGTCGGGCAATGTCATGCGGTATTCGGCTTTGCTTTCCGCCCAAGTTGAAGTGATCCTGTGTGTAAATAATGTCGAATGTTGCCGGTCGGTTGCATAAGCGTTAGTcaattatggccaaagagatctgatttgtggaGTCGAGAAAAAAACCTTCATATAAAACGCGGCCGACATATTATGGCCACCAGTCGTTACCGCGCCACGGTCCACAGAAGAGGATTAAAAAAATATCACACAGCCGAAGGCTAGAGAAGAACCCCCTATAGCTGAACATATATAAACAAATATATTTTTTTTTATTGCCAACACACTTTGGCTTAAGTGTTAAGAGTGATTGTCAGCTTAGAGCTAAGTGCAATGTTCCAGGACAAAGGGTGGATCCTTGTGACCCTGATCACCTTGGTGACGCCGTCTTGGGCTGCTTACAAACTTCAGGAGCGATATAGTTGGAGCCAGCTGGACTTTGCTTTCCCGAATACCCGACTAAAGGACCAAGCTCTGGCTAGTGGAGATTATATTCCGCAAAATGCTCTACCTGTTGGAGTCGAACACTTTGGCAATCGGTTATTCGTCACTGTTCCCCGCTGGCGTGATGGGATTCCGGCCACTCTGACCTATATAAACATGGACCGCAGTTTGACGGGTTCACCGGAGCTAATTCCGTATCCAGATTGGCGCTCAAATACAGCTGGAGATTGCGCCAACAGTATTACCACTGCCTACCGCATTAAAGTGGATGAGTGTGGTCGGCTGTGGGTTTTGGACACTGGAACCGTGGGCATCGGCAATACCACCACTAATCCGTGCTCCTATGCGGTAAATGTCTTTGACTTGACCACGGATACGCGAATTCGGAGATACGAGCTACCTGGCGTGGACACAAATCCAAATACTTTCATAGCTAACATTGCCGTGGATATAGGCAAAAATTGCGATGATGCATATGCCTATTTTGCCGATGAATTGGGATACGGCTTGATTGCTTACTCCTGGGAACTGAACAAGTCCTGGAGATTCTCGGCACATTCGTATTTTTTCCCCGATCCATTGAGGGGCGATTTCAATGTCGCTGGTATTAACTTCCAATGGGGCGAGGAGGGTATATTTGGTATGTCCCTTTCGCCCATTCGATCGGATGGTTATCGTACCCTGTACTTTAGTCCGTTAGCAAGTCATCGACAATTTGCCGTATCCACGAGGATTTTGAGGGATGAAACCAGGACGGAAGATAGCTATCATGACTTTGTTGCCTTAGATGAACGGGGTCCAAACTCCCATACCACTTCACGTGTGATGAGCGATGATGGAATTGAGCTGTTCAATTTAATAGATCAAAATGCAGTGGGTTGCTGGCACTCATCAATGCCGTACTCACCGCAATTTCATGGCATTGTGGATCGCGATGACGTTGGCTTAGTTTTTCCGGCCGATGTGAAAATTGATGAGAACAAAAACGTTTGGGTTCTATCCGATAGGATGCCCGTTTTCTTGCTGTCTGACTTGGATTATTCAGATACTAATTTCCGAATTTACACGGCTCCCTTGGCCACTTTAATTGAGAATACTGTGTGTGATTTGAGGAATAACGCCTATGGGCCGCCAAATACCGTTTCAATACCAAAACAAGCCGTTTTGCCAATGGGTCCACCGTTATATACGAAACAATATCGTCCTGTCTTGCCACAGAAACCTCAGACCAGCTGGGCTTCCTCGCCGCCTCCTCCAAGTCGCACTTATTTGCCCGCCAATTCAGGCAATGTAGTCTCCAGTATTAGTGTCTCTACAAATTCTGTGGGTCCTGCAGGAGTGGAGGTGCCAAAGGCCTATATTTTCAACCAGCACAACGGCATAAATTACGAGACAAGTGGTCCCCATCTATTTCCCACCCATCAACCCGCCCAACCGGGTGGCCAGGATGGTGGGTTAAAAACTTATGTGAATGCCCGCCAATCTGGGTGGTGGCATCATCAGCATCAAGGTTAACATAATCCTACACACGGTACTTGGGTATATTCTCACACACTCGATTGATGTAAAGAATATTTAAAGACAACAACATAGGGCAACAGCGGTTAAAAAAACCACATGACGTATGAGCAAGTGGCAAATCAATACTTTATCTAGTTATGTTAAGCAAAAAATAACAATAAATCAACTTTTTTTTGAAGGTTAAGAGTTTACGCAATTTTCTTGAGCGGAAAAAGCGGAAAAAATGTAAGTATGCATAAATTCTAAATATATCAACAACTGTACATTTTCTGGAGTACTACTACCAGGCAAGAAAGTAGGTTGATAAAGCTATGCACAAGATCTTGTTTGGGTGCAGGGAAAGTTCAACTTAATCGCTCAATTTGAGATCGCCTGGTCGCTTGAGATTCGACTGTAATTGAAATTTTTGCTTTTGATCGGAGCCAGACTTCAGACGGGGCAAACAAAAAGACTTTGTTGGTGGTAGGGTAGGATCCGTTGACCTGCAGGTCTAGTCCTAGGTAGAGGGCCCTTAAGGCGCCTATGGAGCTCTCAGACCGGTTGAGGCGGCCGCTACGCTCGAGGATGGATATCTATGCTAGCGAGATCGATACGTGCATGCGATTCTAGAGATCCACTAGTGTCGACGATGTAGGTCACGGTCTCGAAGCCGCGGTGCGGGTGCCAGGGCGTGCCCTTGGGCTCCCCGGGCGCGTACTCCACCTCACCCATCTGGTCCATCATGATGAACGGGTCGAGGTGGCGGTAGTTGATCCCGGCGAACGCGCGGCGCACCGGGAAGCCCTCGCCCTCGAAACCGCTGGGCGCGGTGGTCACGGTGAGCACGGGACGTGCGACGGCGTCGGCGGGTGCGGATACGCGGGGCAGCGTCAGCGGGTTCTCGACGGTCACGGCGGGCATGTCGACACTAGTTCTAGCCAGCTTTTGTTCCCTTTAGTGAGGGTTAATTTCGAGCTTGGCGTAATCATGGTCATAGCTGTTTCCTGTGTGAAATTGTTATCCGCTCACAATTCCACACAACATACGAGCCGGAAGCATAAAGTGTAAAGCCTGGGGTGCCTAATGAGTGAGCTAACTCACATTAATTGCGTTGCGCTCACTGCCCGCTTTCCAGTCGGGAAACCTGTCGTGCCAGCTGCATTAATGAATCGGCCAACGCGCGGGGAGAGGCGGTTTGCGTATTGGGCGCTCTTCCGCTTCCTCGCTCACTGACTCGCTGCGCTCGGTCGTTCGGCTGCGGCGAGCGGTATCAGCTCACTCAAAGGCGGTAATACGGTTATCCACAGAATCAGGGGATAACGCAGGAAAGAACATGTGAGCAAAAGGCCAGCAAAAGGCCAGGAACCGTAAAAAGGCCGCGTTGCTGGCGTTTTTCCATAGGCTCCGCCCCCCTGACGAGCATCACAAAAATCGACGCTCAAGTCAGAGGTGGCGAAACCCGACAGGACTATAAAGATACCAGGCGTTTCCCCCTGGAAGCTCCCTCGTGCGCTCTCCTGTTCCGACCCTGCCGCTTACCGGATACCTGTCCGCCTTTCTCCCTTCGGGAAGCGTGGCGCTTTCTCATAGCTCACGCTGTAGGTATCTCAGTTCGGTGTAGGTCGTTCGCTCCAAGCTGGGCTGTGTGCACGAACCCCCCGTTCAGCCCGACCGCTGCGCCTTATCCGGTAACTATCGTCTTGAGTCCAACCCGGTAAGACACGACTTATCGCCACTGGCAGCAGCCACTGGTAACAGGATTAGCAGAGCGAGGTATGTAGGCGGTGCTACAGAGTTCTTGAAGTGGTGGCCTAACTACGGCTACACTAGAAGGACAGTATTTGGTATCTGCGCTCTGCTGAAGCCAGTTACCTTCGGAAAAAGAGTTGGTAGCTCTTGATCCGGCAAACAAACCACCGCTGGTAGCGGTGGTTTTTTTGTTTGCAAGCAGCAGATTACGCGCAGAAAAAAAGGATCTCAAGAAGATCCTTTGATCTTTTCTACGGGGTCTGACGCTCAGTGGAACGAAAACTCACGTTAAGGGATTTTGGTCATGAGATTATCAAAAAGGATCTTCACCTAGATCCTTTTAAATTAAAAATGAAGTTTTAAATCAATCTAAAGTATATATGAGTAAACTTGGTCTGACAGTTACCAATGCTTAATCAGTGAGGCACCTATCTCAGCGATCTGTCTATTTCGTTCATCCATAGTTGCCTGACTCCCCGTCGTGTAGATAACTACGATACGGGAGGGCTTACCATCTGGCCCCAGTGCTGCAATGATACCGCGAGACCCACGCTCACCGGCTCCAGATTTATCAGCAATAAACCAGCCAGCCGGAAGGGCCGAGCGCAGAAGTGGTCCTGCAACTTTATCCGCCTCCATCCAGTCTATTAATTGTTGCCGGGAAGCTAGAGTAAGTAGTTCGCCAGTTAATAGTTTGCGCAACGTTGTTGCCATTGCTACAGGCATCGTGGTGTCACGCTCGTCGTTTGGTATGGCTTCATTCAGCTCCGGTTCCCAACGATCAAGGCGAGTTACATGATCCCCCATGTTGTGCAAAAAAGCGGTTAGCTCCTTCGGTCCTCCGATCGTTGTCAGAAGTAAGTTGGCCGCAGTGTTATCACTCATGGTTATGGCAGCACTGCATAATTCTCTTACTGTCATGCCATCCGTAAGATGCTTTTCTGTGACTGGTGAGTACTCAACCAAGTCATTCTGAGAATAGTGTATGCGGCGACCGAGTTGCTCTTGCCCGGCGTCAATACGGGATAATACCGCGCCACATAGCAGAACTTTAAAAGTGCTCATCATTGGAAAACGTTCTTCGGGGCGAAAACTCTCAAGGATCTTACCGCTGTTGAGATCCAGTTCGATGTAACCCACTCGTGCACCCAACTGATCTTCAGCATCTTTTACTTTCACCAGCGTTTCTGGGTGAGCAAAAACAGGAAGGCAAAATGCCGCAAAAAAGGGAATAAGGGCGACACGGAAATGTTGAATACTCATACTCTTCCTTTTTCAATATTATTGAAGCATTTATCAGGGTTATTGTCTCATGAGCGGATACATATTTGAATGTATTTAGAAAAATAAACAAATAGGGGTTCCGCGCACATTTCCCCGAAAAGTGCCAC

y*^wing2+^*

y min promoter+CDS+3‘UTR (bp1680-4014)

MCS: 4014-4130

Sequence of the linker_EGFP_linker donor template

CTAAATTGTAAGCGTTAATATTTTGTTAAAATTCGCGTTAAATTTTTGTTAAATCAGCTCATTTTTTAACCAATAGGCCGAAATCGGCAAAATCCCTTATAAATCAAAAGAATAGACCGAGATAGGGTTGAGTGTTGTTCCAGTTTGGAACAAGAGTCCACTATTAAAGAACGTGGACTCCAACGTCAAAGGGCGAAAAACCGTCTATCAGGGCGATGGCCCACTACGTGAACCATCACCCTAATCAAGTTTTTTGGGGTCGAGGTGCCGTAAAGCACTAAATCGGAACCCTAAAGGGAGCCCCCGATTTAGAGCTTGACGGGGAAAGCCGGCGAACGTGGCGAGAAAGGAAGGGAAGAAAGCGAAAGGAGCGGGCGCTAGGGCGCTGGCAAGTGTAGCGGTCACGCTGCGCGTAACCACCACACCCGCCGCGCTTAATGCGCCGCTACAGGGCGCGTCCCATTCGCCATTCAGGCTGCGCAACTGTTGGGAAGGGCGATCGGTGCGGGCCTCTTCGCTATTACGCCAGCTGGCGAAAGGGGGATGTGCTGCAAGGCGATTAAGTTGGGTAACGCCAGGGTTTTCCCAGTCACGACGTTGTAAAACGACGGCCAGTGAGCGCGCGTAATACGACTCACTATAGGGCGAATTGGAGCTCGAAGACttTCCGGAGGTTCCGGTGGAAGCGGAGGTAGCGGCggatccGTGTCCAAGGGCGAGGAGCTGTTCACCGGCGTGGTGCCCATCCTGGTGGAGCTGGATGGCGACGTGAACGGCCACAAGTTCAGCGTGCGCGGCGAGGGCGAGGGCGACGCCACCAACGGCAAGCTGACCCTGAAGTTCATCTGCACCACCGGCAAGCTGCCCGTGCCCTGGCCCACCCTGGTGACCACCCTGACCTACGGCGTGCAGTGCTTCAGCCGCTACCCCGATCACATGAAGCAGCACGATTTCTTCAAGAGCGCCATGCCCGAGGGCTACGTGCAGGAGCGCACCATCAGCTTCAAGGATGACGGCACCTACAAGACCCGCGCCGAGGTGAAGTTCGAGGGCGATACCCTGGTGAACCGCATCGAGCTGAAGGGCATCGATTTCAAGGAGGATGGCAACATCCTGGGCCACAAGCTGGAGTACAACTTCAACAGCCACAACGTGTACATCACCGCCGATAAGCAGAAGAACGGCATCAAGGCCAACTTCAAGATCCGCCACAATGTGGAGGATGGCTCCGTGCAGCTGGCCGATCACTACCAGCAGAACACCCCCATCGGCGACGGCCCAGTGCTGCTGCCCGATAACCACTACCTGAGCACCCAGAGCGTGCTGTCCAAGGACCCCAACGAGAAGCGCGATCACATGGTGCTGCTGGAGTTCGTGACCGCCGCCGGCATCACCCTGGGCATGGATGAGCTGTACAAGGGCGGATCCGGAGGTAGCGGTGGAAGCGGAGGTTCTccGTCTTCGATATCAAGCTTATCGATACCGTCGACCTCGAGGGGGGGCCCGGTACCCAGCTTTTGTTCCCTTTAGTGAGGGTTAATTGCGCGCTTGGCGTAATCATGGTCATAGCTGTTTCCTGTGTGAAATTGTTATCCGCTCACAATTCCACACAACATACGAGCCGGAAGCATAAAGTGTAAAGCCTGGGGTGCCTAATGAGTGAGCTAACTCACATTAATTGCGTTGCGCTCACTGCCCGCTTTCCAGTCGGGAAACCTGTCGTGCCAGCTGCATTAATGAATCGGCCAACGCGCGGGGAGAGGCGGTTTGCGTATTGGGCGCTCTTCCGCTTCCTCGCTCACTGACTCGCTGCGCTCGGTCGTTCGGCTGCGGCGAGCGGTATCAGCTCACTCAAAGGCGGTAATACGGTTATCCACAGAATCAGGGGATAACGCAGGAAAGAACATGTGAGCAAAAGGCCAGCAAAAGGCCAGGAACCGTAAAAAGGCCGCGTTGCTGGCGTTTTTCCATAGGCTCCGCCCCCCTGACGAGCATCACAAAAATCGACGCTCAAGTCAGAGGTGGCGAAACCCGACAGGACTATAAAGATACCAGGCGTTTCCCCCTGGAAGCTCCCTCGTGCGCTCTCCTGTTCCGACCCTGCCGCTTACCGGATACCTGTCCGCCTTTCTCCCTTCGGGAAGCGTGGCGCTTTCTCATAGCTCACGCTGTAGGTATCTCAGTTCGGTGTAGGTCGTTCGCTCCAAGCTGGGCTGTGTGCACGAACCCCCCGTTCAGCCCGACCGCTGCGCCTTATCCGGTAACTATCGTCTTGAGTCCAACCCGGTAAGACACGACTTATCGCCACTGGCAGCAGCCACTGGTAACAGGATTAGCAGAGCGAGGTATGTAGGCGGTGCTACAGAGTTCTTGAAGTGGTGGCCTAACTACGGCTACACTAGAAGGACAGTATTTGGTATCTGCGCTCTGCTGAAGCCAGTTACCTTCGGAAAAAGAGTTGGTAGCTCTTGATCCGGCAAACAAACCACCGCTGGTAGCGGTGGTTTTTTTGTTTGCAAGCAGCAGATTACGCGCAGAAAAAAAGGATCTCAAGAAGATCCTTTGATCTTTTCTACGGGGTCTGACGCTCAGTGGAACGAAAACTCACGTTAAGGGATTTTGGTCATGAGATTATCAAAAAGGATCTTCACCTAGATCCTTTTAAATTAAAAATGAAGTTTTAAATCAATCTAAAGTATATATGAGTAAACTTGGTCTGACAGTTACCAATGCTTAATCAGTGAGGCACCTATCTCAGCGATCTGTCTATTTCGTTCATCCATAGTTGCCTGACTCCCCGTCGTGTAGATAACTACGATACGGGAGGGCTTACCATCTGGCCCCAGTGCTGCAATGATACCGCGtGACCCACGCTCACCGGCTCCAGATTTATCAGCAATAAACCAGCCAGCCGGAAGGGCCGAGCGCAGAAGTGGTCCTGCAACTTTATCCGCCTCCATCCAGTCTATTAATTGTTGCCGGGAAGCTAGAGTAAGTAGTTCGCCAGTTAATAGTTTGCGCAACGTTGTTGCCATTGCTACAGGCATCGTGGTGTCACGCTCGTCGTTTGGTATGGCTTCATTCAGCTCCGGTTCCCAACGATCAAGGCGAGTTACATGATCCCCCATGTTGTGCAAAAAAGCGGTTAGCTCCTTCGGTCCTCCGATCGTTGTCAGAAGTAAGTTGGCCGCAGTGTTATCACTCATGGTTATGGCAGCACTGCATAATTCTCTTACTGTCATGCCATCCGTAAGATGCTTTTCTGTGACTGGTGAGTACTCAACCAAGTCATTCTGAGAATAGTGTATGCGGCGACCGAGTTGCTCTTGCCCGGCGTCAATACGGGATAATACCGCGCCACATAGCAGAACTTTAAAAGTGCTCATCATTGGAAAACGTTCTTCGGGGCGAAAACTCTCAAGGATCTTACCGCTGTTGAGATCCAGTTCGATGTAACCCACTCGTGCACCCAACTGATCTTCAGCATCTTTTACTTTCACCAGCGTTTCTGGGTGAGCAAAAACAGGAAGGCAAAATGCCGCAAAAAAGGGAATAAGGGCGACACGGAAATGTTGAATACTCATACTCTTCCTTTTTCAATATTATTGAAGCATTTATCAGGGTTATTGTCTCATGAGCGGATACATATTTGAATGTATTTAGAAAAATAAACAAATAGGGGTTCCGCGCACATTTCCCCGAAAAGTGCCAC

Sequence of the Linker_mCherry_Linker donor template

CTAAATTGTAAGCGTTAATATTTTGTTAAAATTCGCGTTAAATTTTTGTTAAATCAGCTCATTTTTTAACCAATAGGCCGAAATCGGCAAAATCCCTTATAAATCAAAAGAATAGACCGAGATAGGGTTGAGTGTTGTTCCAGTTTGGAACAAGAGTCCACTATTAAAGAACGTGGACTCCAACGTCAAAGGGCGAAAAACCGTCTATCAGGGCGATGGCCCACTACGTGAACCATCACCCTAATCAAGTTTTTTGGGGTCGAGGTGCCGTAAAGCACTAAATCGGAACCCTAAAGGGAGCCCCCGATTTAGAGCTTGACGGGGAAAGCCGGCGAACGTGGCGAGAAAGGAAGGGAAGAAAGCGAAAGGAGCGGGCGCTAGGGCGCTGGCAAGTGTAGCGGTCACGCTGCGCGTAACCACCACACCCGCCGCGCTTAATGCGCCGCTACAGGGCGCGTCCCATTCGCCATTCAGGCTGCGCAACTGTTGGGAAGGGCGATCGGTGCGGGCCTCTTCGCTATTACGCCAGCTGGCGAAAGGGGGATGTGCTGCAAGGCGATTAAGTTGGGTAACGCCAGGGTTTTCCCAGTCACGACGTTGTAAAACGACGGCCAGTGAGCGCGCGTAATACGACTCACTATAGGGCGAATTGGAGCTCCACCgcggTGGGAAGACttTCCGGAGGTTCCGGTGGAAGCGGAGGTAGCGGCggatccGTGAGCAAGGGCGAGGAGGATAACATGGCCATCATCAAGGAGTTCATGCGCTTCAAGGTGCACATGGAGGGCTCCGTGAACGGCCACGAGTTCGAGATCGAGGGCGAGGGCGAGGGCCGCCCCTACGAGGGCACCCAGACCGCCAAGCTGAAGGTGACCAAGGGTGGCCCCCTGCCCTTCGCCTGGGACATCCTGTCCCCTCAGTTCATGTACGGCTCCAAGGCCTACGTGAAGCACCCCGCCGACATCCCCGACTACTTGAAGCTGTCCTTCCCCGAGGGCTTCAAGTGGGAGCGCGTGATGAACTTCGAGGACGGCGGCGTGGTGACCGTGACCCAGGACTCCTCCCTGCAGGACGGCGAGTTCATCTACAAGGTGAAGCTGCGCGGCACCAACTTCCCCTCCGACGGCCCCGTAATGCAGAAGAAAACCATGGGCTGGGAGGCCTCCTCCGAGCGGATGTACCCCGAGGACGGCGCCCTGAAGGGCGAGATCAAGCAGAGGCTGAAGCTGAAGGACGGCGGCCACTACGACGCTGAGGTCAAGACCACCTACAAGGCCAAGAAGCCCGTGCAGCTGCCCGGCGCCTACAACGTCAACATCAAGTTGGACATCACCTCCCACAACGAGGACTACACCATCGTGGAACAGTACGAACGCGCCGAGGGCCGCCACTCCACCGGCGGCATGGACGAGCTGTACAAGggcgGATCCGGAGGTAGCGGTGGAAGCGGAGGTTCTccGTCTTCGATATCAAGCTTATCGATACCGTCGACCTCGAGGGGGGGCCCGGTACCCAGCTTTTGTTCCCTTTAGTGAGGGTTAATTGCGCGCTTGGCGTAATCATGGTCATAGCTGTTTCCTGTGTGAAATTGTTATCCGCTCACAATTCCACACAACATACGAGCCGGGAGCATAAAGTGTAAAGCCTGGGGTGCCTAATGAGTGAGCTAACTCACATTAATTGCGTTGCGCTCACTGCCCGCTTTCCAGTCGGGAAACCTGTCGTGCCAGCTGCATTAATGAATCGGCCAACGCGCGGGGAGAGGCGGTTTGCGTATTGGGCGCTCTTCCGCTTCCTCGCTCACTGACTCGCTGCGCTCGGTCGTTCGGCTGCGGCGAGCGGTATCAGCTCACTCAAAGGCGGTAATACGGTTATCCACAGAATCAGGGGATAACGCAGGAAAGAACATGTGAGCAAAAGGCCAGCAAAAGGCCAGGAACCGTAAAAAGGCCGCGTTGCTGGCGTTTTTCCATAGGCTCCGCCCCCCTGACGAGCATCACAAAAATCGACGCTCAAGTCAGAGGTGGCGAAACCCGACAGGACTATAAAGATACCAGGCGTTTCCCCCTGGAAGCTCCCTCGTGCGCTCTCCTGTTCCGACCCTGCCGCTTACCGGATACCTGTCCGCCTTTCTCCCTTCGGGAAGCGTGGCGCTTTCTCATAGCTCACGCTGTAGGTATCTCAGTTCGGTGTAGGTCGTTCGCTCCAAGCTGGGCTGTGTGCACGAACCCCCCGTTCAGCCCGACCGCTGCGCCTTATCCGGTAACTATCGTCTTGAGTCCAACCCGGTAAGACACGACTTATCGCCACTGGCAGCAGCCACTGGTAACAGGATTAGCAGAGCGAGGTATGTAGGCGGTGCTACAGAGTTCTTGAAGTGGTGGCCTAACTACGGCTACACTAGAAGGACAGTATTTGGTATCTGCGCTCTGCTGAAGCCAGTTACCTTCGGAAAAAGAGTTGGTAGCTCTTGATCCGGCAAACAAACCACCGCTGGTAGCGGTGGTTTTTTTGTTTGCAAGCAGCAGATTACGCGCAGAAAAAAAGGATCTCAAGAAGATCCTTTGATCTTTTCTACGGGGTCTGACGCTCAGTGGAACGAAAACTCACGTTAAGGGATTTTGGTCATGAGATTATCAAAAAGGATCTTCACCTAGATCCTTTTAAATTAAAAATGAAGTTTTAAATCAATCTAAAGTATATATGAGTAAACTTGGTCTGACAGTTACCAATGCTTAATCAGTGAGGCACCTATCTCAGCGATCTGTCTATTTCGTTCATCCATAGTTGCCTGACTCCCCGTCGTGTAGATAACTACGATACGGGAGGGCTTACCATCTGGCCCCAGTGCTGCAATGATACCGCGtGACCCACGCTCACCGGCTCCAGATTTATCAGCAATAAACCAGCCAGCCGGAAGGGCCGAGCGCAGAAGTGGTCCTGCAACTTTATCCGCCTCCATCCAGTCTATTAATTGTTGCCGGGAAGCTAGAGTAAGTAGTTCGCCAGTTAATAGTTTGCGCAACGTTGTTGCCATTGCTACAGGCATCGTGGTGTCACGCTCGTCGTTTGGTATGGCTTCATTCAGCTCCGGTTCCCAACGATCAAGGCGAGTTACATGATCCCCCATGTTGTGCAAAAAAGCGGTTAGCTCCTTCGGTCCTCCGATCGTTGTCAGAAGTAAGTTGGCCGCAGTGTTATCACTCATGGTTATGGCAGCACTGCATAATTCTCTTACTGTCATGCCATCCGTAAGATGCTTTTCTGTGACTGGTGAGTACTCAACCAAGTCATTCTGAGAATAGTGTATGCGGCGACCGAGTTGCTCTTGCCCGGCGTCAATACGGGATAATACCGCGCCACATAGCAGAACTTTAAAAGTGCTCATCATTGGAAAACGTTCTTCGGGGCGAAAACTCTCAAGGATCTTACCGCTGTTGAGATCCAGTTCGATGTAACCCACTCGTGCACCCAACTGATCTTCAGCATCTTTTACTTTCACCAGCGTTTCTGGGTGAGCAAAAACAGGAAGGCAAAATGCCGCAAAAAAGGGAATAAGGGCGACACGGAAATGTTGAATACTCATACTCTTCCTTTTTCAATATTATTGAAGCATTTATCAGGGTTATTGTCTCATGAGCGGATACATATTTGAATGTATTTAGAAAAATAAACAAATAGGGGTTCCGCGCACATTTCCCCGAAAAGTGCCAC

Sequence of the T2A_Gal4 (No ATG, NoStop codon) donor template

CTAAATTGTAAGCGTTAATATTTTGTTAAAATTCGCGTTAAATTTTTGTTAAATCAGCTCATTTTTTAACCAATAGGCCGAAATCGGCAAAATCCCTTATAAATCAAAAGAATAGACCGAGATAGGGTTGAGTGTTGTTCCAGTTTGGAACAAGAGTCCACTATTAAAGAACGTGGACTCCAACGTCAAAGGGCGAAAAACCGTCTATCAGGGCGATGGCCCACTACGTGAACCATCACCCTAATCAAGTTTTTTGGGGTCGAGGTGCCGTAAAGCACTAAATCGGAACCCTAAAGGGAGCCCCCGATTTAGAGCTTGACGGGGAAAGCCGGCGAACGTGGCGAGAAAGGAAGGGAAGAAAGCGAAAGGAGCGGGCGCTAGGGCGCTGGCAAGTGTAGCGGTCACGCTGCGCGTAACCACCACACCCGCCGCGCTTAATGCGCCGCTACAGGGCGCGTCCCATTCGCCATTCAGGCTGCGCAACTGTTGGGAAGGGCGATCGGTGCGGGCCTCTTCGCTATTACGCCAGCTGGCGAAAGGGGGATGTGCTGCAAGGCGATTAAGTTGGGTAACGCCAGGGTTTTCCCAGTCACGACGTTGTAAAACGACGGCCAGTGAGCGCGCGTAATACGACTCACTATAGGGCGAATTGGAGCTCGAAGACttgagggccgcggcagcctgctgacctgcggcgatgtggaggagaaccccgggcccatgaagctgctgagcagcatcgagcaggcctgcgatatctgccgcctgaagaagctgaagtgcagcaaggagaagcccaagtgcgccaagtgcctgaagaacaactgggagtgccgctacagccccaagaccaagcgcagccccctgacccgcgcccacctgaccgaggtggagagccgcctggagcgcctggagcagctgttcctgctgatcttcccccgcgaggatctggatatgatcctgaagatggatagcctgcaggatatcaaggccctgctgaccggcctgttcgtgcaggataacgtgaacaaggatgccgtgaccgatcgcctggccagcgtggaaaccgatatgcccctgaccctgcgccagcaccgcatcagcgccaccagcagcagcgaggagagcagcaacaagggccagcgccagctgaccgtgagcatcgatagcgccgcccaccacgataacagcaccatccccctggatttcatgccccgcgatgccctgcacggcttcgattggagcgaggaggatgatatgagcgatggcctgcccttcctgaaaaccgatcccaacaacaacggcttcttcggcgatggcagcctgctgtgcatcctgcgcagcatcggcttcaagcccgagaactacaccaacagcaacgtgaaccgcctgcccaccatgatcaccgatcgctacaccctggccagccgcagcaccaccagccgcctgctgcagagctacctgaacaacttccacccctactgccccatcgtgcacagccccaccctgatgatgctgtacaacaaccagatcgagatcgccagcaaggatcagtggcagatcctgttcaactgcatcctggccatcggcgcctggtgcatcgagggcgagagcaccgatatcgatgtgttctactaccagaacgccaagagccacctgaccagcaaggtgttcgagagcggcagcatcatcctggtgaccgccctgcacctgctgagccgctacacccagtggcgccaaaagaccaacaccagctacaacttccacagcttcagcatccgcatggccatcagcctgggcctgaaccgcgatctgcccagcagcttcagcgatagcagcatcctggagcagcgccgccgcatctggtggagcgtgtacagctgggagatccagctgagcctgctgtacggccgcagcatccagctgagccagaacaccatcagcttccccagcagcgtggatgatgtgcagcgcaccaccaccggccccaccatctaccacggcatcatcgagactgcccgcctgctgcaggtgttcaccaagatctacgagctggataagaccgtgaccgccgagaagagccccatctgcgccaagaagtgcctgatgatctgcaacgagatcgaggaggtgagccgccaggcccccaagttcctgcagatggatatcagcaccaccgccctgaccaacctgctgaaggagcacccctggctgagcttcacccgcttcgagctgaagtggaagcagctgagcctgatcatctacgtgctgcgcgatttcttcaccaacttcacccagaagaagagccagctggagcaggatcagaacgatcaccagagctacgaggtgaagcgctgcagcatcatgctgagcgatgccgcccagcgcaccgtgatgagcgtgagcagctacatggataaccacaacgtgaccccctacttcgcctggaactgcagctactacctgttcaacgccgtgctggtgcccatcaagaccctgctgagcaacagcaagagcaacgccgagaacaacgaaacggcccagctgctgcagcagatcaacaccgtgctgatgctgctgaagaagctggccaccttcaagatccagacctgcgagaagtacatccaggtgctggaggaggtgtgcgcccccttcctgctgagccagtgcgccatccccctgccccacatcagctacaacaacagcaacggcagcgccatcaagaacatcgtgggcagcgccaccatcgcccagtaccccaccctgcccgaggagaacgtgaacaacatcagcgtgaagtacgtgagccccggcagcgtgggacccagccccgtgcccctgaagagcggcgccagcttcagcgatctggtgaagctgctgagcaaccgcccccccagccgcaacagccccgtgaccatcccccgcagcacccccagccaccgcagcgtgacccccttcctgggccagcagcagcagctgcagagcctggtgcccctgacccccagcgccctgttcggcggcgccaacttcaaccagagcggcaacatcgccgatagcagcctgagcttcaccttcaccaacagcagcaacggccccaacctgatcaccacccagaccaacagccaggccctgagccagcccatcgccagcagcaacgtgcacgataacttcatgaacaacgagatcaccgccagcaagatcgatgatggcaacaacagcaagcccctgagccccggctggaccgatcagaccgcctacaacgccttcggcatcaccaccggcatgttcaacaccaccaccatggatgatgtgtacaactacctgttcgatgatgaggataccccccccaaccccaagaaggagccGTCTTCGATATCAAGCTTATCGATACCGTCGACCTCGAGGGGGGGCCCGGTACCCAGCTTTTGTTCCCTTTAGTGAGGGTTAATTGCGCGCTTGGCGTAATCATGGTCATAGCTGTTTCCTGTGTGAAATTGTTATCCGCTCACAATTCCACACAACATACGAGCCGGGAGCATAAAGTGTAAAGCCTGGGGTGCCTAATGAGTGAGCTAACTCACATTAATTGCGTTGCGCTCACTGCCCGCTTTCCAGTCGGGAAACCTGTCGTGCCAGCTGCATTAATGAATCGGCCAACGCGCGGGGAGAGGCGGTTTGCGTATTGGGCGCTCTTCCGCTTCCTCGCTCACTGACTCGCTGCGCTCGGTCGTTCGGCTGCGGCGAGCGGTATCAGCTCACTCAAAGGCGGTAATACGGTTATCCACAGAATCAGGGGATAACGCAGGAAAGAACATGTGAGCAAAAGGCCAGCAAAAGGCCAGGAACCGTAAAAAGGCCGCGTTGCTGGCGTTTTTCCATAGGCTCCGCCCCCCTGACGAGCATCACAAAAATCGACGCTCAAGTCAGAGGTGGCGAAACCCGACAGGACTATAAAGATACCAGGCGTTTCCCCCTGGAAGCTCCCTCGTGCGCTCTCCTGTTCCGACCCTGCCGCTTACCGGATACCTGTCCGCCTTTCTCCCTTCGGGAAGCGTGGCGCTTTCTCATAGCTCACGCTGTAGGTATCTCAGTTCGGTGTAGGTCGTTCGCTCCAAGCTGGGCTGTGTGCACGAACCCCCCGTTCAGCCCGACCGCTGCGCCTTATCCGGTAACTATCGTCTTGAGTCCAACCCGGTAAGACACGACTTATCGCCACTGGCAGCAGCCACTGGTAACAGGATTAGCAGAGCGAGGTATGTAGGCGGTGCTACAGAGTTCTTGAAGTGGTGGCCTAACTACGGCTACACTAGAAGGACAGTATTTGGTATCTGCGCTCTGCTGAAGCCAGTTACCTTCGGAAAAAGAGTTGGTAGCTCTTGATCCGGCAAACAAACCACCGCTGGTAGCGGTGGTTTTTTTGTTTGCAAGCAGCAGATTACGCGCAGAAAAAAAGGATCTCAAGAAGATCCTTTGATCTTTTCTACGGGGTCTGACGCTCAGTGGAACGAAAACTCACGTTAAGGGATTTTGGTCATGAGATTATCAAAAAGGATCTTCACCTAGATCCTTTTAAATTAAAAATGAAGTTTTAAATCAATCTAAAGTATATATGAGTAAACTTGGTCTGACAGTTACCAATGCTTAATCAGTGAGGCACCTATCTCAGCGATCTGTCTATTTCGTTCATCCATAGTTGCCTGACTCCCCGTCGTGTAGATAACTACGATACGGGAGGGCTTACCATCTGGCCCCAGTGCTGCAATGATACCGCGtGACCCACGCTCACCGGCTCCAGATTTATCAGCAATAAACCAGCCAGCCGGAAGGGCCGAGCGCAGAAGTGGTCCTGCAACTTTATCCGCCTCCATCCAGTCTATTAATTGTTGCCGGGAAGCTAGAGTAAGTAGTTCGCCAGTTAATAGTTTGCGCAACGTTGTTGCCATTGCTACAGGCATCGTGGTGTCACGCTCGTCGTTTGGTATGGCTTCATTCAGCTCCGGTTCCCAACGATCAAGGCGAGTTACATGATCCCCCATGTTGTGCAAAAAAGCGGTTAGCTCCTTCGGTCCTCCGATCGTTGTCAGAAGTAAGTTGGCCGCAGTGTTATCACTCATGGTTATGGCAGCACTGCATAATTCTCTTACTGTCATGCCATCCGTAAGATGCTTTTCTGTGACTGGTGAGTACTCAACCAAGTCATTCTGAGAATAGTGTATGCGGCGACCGAGTTGCTCTTGCCCGGCGTCAATACGGGATAATACCGCGCCACATAGCAGAACTTTAAAAGTGCTCATCATTGGAAAACGTTCTTCGGGGCGAAAACTCTCAAGGATCTTACCGCTGTTGAGATCCAGTTCGATGTAACCCACTCGTGCACCCAACTGATCTTCAGCATCTTTTACTTTCACCAGCGTTTCTGGGTGAGCAAAAACAGGAAGGCAAAATGCCGCAAAAAAGGGAATAAGGGCGACACGGAAATGTTGAATACTCATACTCTTCCTTTTTCAATATTATTGAAGCATTTATCAGGGTTATTGTCTCATGAGCGGATACATATTTGAATGTATTTAGAAAAATAAACAAATAGGGGTTCCGCGCACATTTCCCCGAAAAGTGCCAC

Sequence of the T2A_Gal4_polyA donor template

ctaaattgtaagcgttaatattttgttaaaattcgcgttaaatttttgttaaatcagctcattttttaaccaataggccgaaatcggcaaaatcccttataaatcaaaagaatagaccgagatagggttgagtgttgttccagtttggaacaagagtccactattaaagaacgtggactccaacgtcaaagggcgaaaaaccgtctatcagggcgatggcccactacgtgaaccatcaccctaatcaagttttttggggtcgaggtgccgtaaagcactaaatcggaaccctaaagggagcccccgatttagagcttgacggggaaagccggcgaacgtggcgagaaaggaagggaagaaagcgaaaggagcgggcgctagggcgctggcaagtgtagcggtcacgctgcgcgtaaccaccacacccgccgcgcttaatgcgccgctacagggcgcgtcccattcgccattcaggctgcgcaactgttgggaagggcgatcggtgcgggcctcttcgctattacgccagctggcgaaagggggatgtgctgcaaggcgattaagttgggtaacgccagggttttcccagtcacgacgttgtaaaacgacggccagtgagcgcgcgtaatacgactcactatagggcgaattggagctccaccgcggtggcgtctctaaacggtctctaaacgaagacttaaacatgcatgtactgacggacacaccgaagccccggcggcaaccctcagcggatgccccggggcttcacgttttcccaggtcagaagcggttttcgggagtagtgccccaactggggtaacctttgagttctctcagttgggggcgtagggccgccgacatgacacaaggggttgtgaccggggtggacacgtacgcgggtgcttacgaccgtcagtcgcgcgagcgcgagaagttcctattctctagaaagtataggaacttcgaattgagtcgatccaacatggcgacttgtcccatccccggcatgtttaaatatactaattattcttgaactaattttaatcaaccgatttatctctcttccgcaggtgggaggttccggtggaagcggaggtagcggcaccggtgcatgcgagggccgcggcagcctgctgacctgcggcgatgtggaggagaaccccgggcccatgaagctgctgagcagcatcgagcaggcctgcgatatctgccgcctgaagaagctgaagtgcagcaaggagaagcccaagtgcgccaagtgcctgaagaacaactgggagtgccgctacagccccaagaccaagcgcagccccctgacccgcgcccacctgaccgaggtggagagccgcctggagcgcctggagcagctgttcctgctgatcttcccccgcgaggatctggatatgatcctgaagatggatagcctgcaggatatcaaggccctgctgaccggcctgttcgtgcaggataacgtgaacaaggatgccgtgaccgatcgcctggccagcgtggaaaccgatatgcccctgaccctgcgccagcaccgcatcagcgccaccagcagcagcgaggagagcagcaacaagggccagcgccagctgaccgtgagcatcgatagcgccgcccaccacgataacagcaccatccccctggatttcatgccccgcgatgccctgcacggcttcgattggagcgaggaggatgatatgagcgatggcctgcccttcctgaaaaccgatcccaacaacaacggcttcttcggcgatggcagcctgctgtgcatcctgcgcagcatcggcttcaagcccgagaactacaccaacagcaacgtgaaccgcctgcccaccatgatcaccgatcgctacaccctggccagccgcagcaccaccagccgcctgctgcagagctacctgaacaacttccacccctactgccccatcgtgcacagccccaccctgatgatgctgtacaacaaccagatcgagatcgccagcaaggatcagtggcagatcctgttcaactgcatcctggccatcggcgcctggtgcatcgagggcgagagcaccgatatcgatgtgttctactaccagaacgccaagagccacctgaccagcaaggtgttcgagagcggcagcatcatcctggtgaccgccctgcacctgctgagccgctacacccagtggcgccaaaagaccaacaccagctacaacttccacagcttcagcatccgcatggccatcagcctgggcctgaaccgcgatctgcccagcagcttcagcgatagcagcatcctggagcagcgccgccgcatctggtggagcgtgtacagctgggagatccagctgagcctgctgtacggccgcagcatccagctgagccagaacaccatcagcttccccagcagcgtggatgatgtgcagcgcaccaccaccggccccaccatctaccacggcatcatcgagactgcccgcctgctgcaggtgttcaccaagatctacgagctggataagaccgtgaccgccgagaagagccccatctgcgccaagaagtgcctgatgatctgcaacgagatcgaggaggtgagccgccaggcccccaagttcctgcagatggatatcagcaccaccgccctgaccaacctgctgaaggagcacccctggctgagcttcacccgcttcgagctgaagtggaagcagctgagcctgatcatctacgtgctgcgcgatttcttcaccaacttcacccagaagaagagccagctggagcaggatcagaacgatcaccagagctacgaggtgaagcgctgcagcatcatgctgagcgatgccgcccagcgcaccgtgatgagcgtgagcagctacatggataaccacaacgtgaccccctacttcgcctggaactgcagctactacctgttcaacgccgtgctggtgcccatcaagaccctgctgagcaacagcaagagcaacgccgagaacaacgaaacggcccagctgctgcagcagatcaacaccgtgctgatgctgctgaagaagctggccaccttcaagatccagacctgcgagaagtacatccaggtgctggaggaggtgtgcgcccccttcctgctgagccagtgcgccatccccctgccccacatcagctacaacaacagcaacggcagcgccatcaagaacatcgtgggcagcgccaccatcgcccagtaccccaccctgcccgaggagaacgtgaacaacatcagcgtgaagtacgtgagccccggcagcgtgggacccagccccgtgcccctgaagagcggcgccagcttcagcgatctggtgaagctgctgagcaaccgcccccccagccgcaacagccccgtgaccatcccccgcagcacccccagccaccgcagcgtgacccccttcctgggccagcagcagcagctgcagagcctggtgcccctgacccccagcgccctgttcggcggcgccaacttcaaccagagcggcaacatcgccgatagcagcctgagcttcaccttcaccaacagcagcaacggccccaacctgatcaccacccagaccaacagccaggccctgagccagcccatcgccagcagcaacgtgcacgataacttcatgaacaacgagatcaccgccagcaagatcgatgatggcaacaacagcaagcccctgagccccggctggaccgatcagaccgcctacaacgccttcggcatcaccaccggcatgttcaacaccaccaccatggatgatgtgtacaactacctgttcgatgatgaggataccccccccaaccccaagaaggagtaataaggcgcgcccaaagatccagacatgataagatacattgatgagtttggacaaaccacaactagaatgcagtgaaaaaaatgctttatttgtgaaatttgtgatgctattgctttatttgtaaccattataagctgcaataaacaagttaacaacaacaattgcattcattttatgtttcaggttcagggggaggtgtgggaggttttttaaagcaagtaaaacctctacaaatgtggtatggctgattatgatcataattcgagctcgcccggggatctaattcaattagagactaattcaattagagctaattcaattaggatccaagcttatcgatttcgaaccctcgaccgccggagtataaatagaggcgcttcgtctacggagcgacaattcaattcaaacaagcaaagtgaacacgtcgctaagcgaaagctaagcaaataaacaagcgcagctgaacaagctaaacaatcggactagagccggtcgccggccggccaccatggtgtccaagggcgaggagctgttcaccggcgtggtgccaattctggtggagctggatggcgacgtgaacggccacaagttcagcgtgtccggcgagggcgagggcgacgccacctatggaaagctgaccctgaagttcatctgcaccaccggcaagctgcccgtgccatggccaaccctcgtgaccaccctgacctatggcgtgcagtgcttcagccgctaccccgatcacatgaagcagcacgatttcttcaagagcgccatgcccgagggctacgtgcaggagcgcaccatctttttcaaggatgacggcaactacaagacccgcgccgaagtgaagttcgagggcgataccctcgtgaaccgcatcgagctgaagggcatcgatttcaaggaggatggaaacatcctgggccacaagctggagtacaactacaacagccacaacgtgtacatcatggccgacaagcagaagaacggcatcaaggccaacttcaagatccgccacaacatcgaggatggcggcgtgcagctggccgatcactaccagcagaacaccccaatcggcgacggcccagtgctgctgcccgataaccattacctgagcacccagagcgccctgagcaaggatcccaacgagaagcgcgaccacatggtgctgctggagtttgtgaccgccgccggcattaccctgggcatggatgagctgtacaagtaggatccagacatgataagatacattgatgagtttggacaaaccacaactagaatgcagtgaaaaaaatgctttatttgtgaaatttgtgatgctattgctttatttgtaaccattataagctgcaataaacaagttaacaacaacaattgcattcattttatgtttcaggttcagggggaggtgtgggaggttttttaaagcaagtaaaacctctacaaatgtggtatggctgattatgatcagaagttcctattctctagaaagtataggaacttctcgcgctcgcgcgactgacggtcgtaagcacccgcgtacgtgtccaccccggtcacaaccccttgtgtcatgtcggcggccctacgcccccaactgagagaactcaaaggttaccccagttggggcactactcccgaaaaccgcttctgacctgggaaaacgtgaagccccggggcatccgctgagggttgccgccggggcttcggtgtgtccgtcagtacgcatgccgcgttgtcttccgcgtgagacccgcgtgagacggatatcaagcttatcgataccgtcgacctcgagggggggcccggtacccagcttttgttccctttagtgagggttaattgcgcgcttggcgtaatcatggtcatagctgtttcctgtgtgaaattgttatccgctcacaattccacacaacatacgagccggaagcataaagtgtaaagcctggggtgcctaatgagtgagctaactcacattaattgcgttgcgctcactgcccgctttccagtcgggaaacctgtcgtgccagctgcattaatgaatcggccaacgcgcggggagaggcggtttgcgtattgggcgctcttccgcttcctcgctcactgactcgctgcgctcggtcgttcggctgcggcgagcggtatcagctcactcaaaggcggtaatacggttatccacagaatcaggggataacgcaggaaagaacatgtgagcaaaaggccagcaaaaggccaggaaccgtaaaaaggccgcgttgctggcgtttttccataggctccgcccccctgacgagcatcacaaaaatcgacgctcaagtcagaggtggcgaaacccgacaggactataaagataccaggcgtttccccctggaagctccctcgtgcgctctcctgttccgaccctgccgcttaccggatacctgtccgcctttctcccttcgggaagcgtggcgctttctcatagctcacgctgtaggtatctcagttcggtgtaggtcgttcgctccaagctgggctgtgtgcacgaaccccccgttcagcccgaccgctgcgccttatccggtaactatcgtcttgagtccaacccggtaagacacgacttatcgccactggcagcagccactggtaacaggattagcagagcgaggtatgtaggcggtgctacagagttcttgaagtggtggcctaactacggctacactagaaggacagtatttggtatctgcgctctgctgaagccagttaccttcggaaaaagagttggtagctcttgatccggcaaacaaaccaccgctggtagcggtggtttttttgtttgcaagcagcagattacgcgcagaaaaaaaggatctcaagaagatcctttgatcttttctacggggtctgacgctcagtggaacgaaaactcacgttaagggattttggtcatgagattatcaaaaaggatcttcacctagatccttttaaattaaaaatgaagttttaaatcaatctaaagtatatatgagtaaacttggtctgacagttaccaatgcttaatcagtgaggcacctatctcagcgatctgtctatttcgttcatccatagttgcctgactccccgtcgtgtagataactacgatacgggagggcttaccatctggccccagtgctgcaatgataccgcgtgacccacgctcaccggctccagatttatcagcaataaaccagccagccggaagggccgagcgcagaagtggtcctgcaactttatccgcctccatccagtctattaattgttgccgggaagctagagtaagtagttcgccagttaatagtttgcgcaacgttgttgccattgctacaggcatcgtggtgtcacgctcgtcgtttggtatggcttcattcagctccggttcccaacgatcaaggcgagttacatgatcccccatgttgtgcaaaaaagcggttagctccttcggtcctccgatcgttgtcagaagtaagttggccgcagtgttatcactcatggttatggcagcactgcataattctcttactgtcatgccatccgtaagatgcttttctgtgactggtgagtactcaaccaagtcattctgagaatagtgtatgcggcgaccgagttgctcttgcccggcgtcaatacgggataataccgcgccacatagcagaactttaaaagtgctcatcattggaaaacgttcttcggggcgaaaactctcaaggatcttaccgctgttgagatccagttcgatgtaacccactcgtgcacccaactgatcttcagcatcttttactttcaccagcgtttctgggtgagcaaaaacaggaaggcaaaatgccgcaaaaaagggaataagggcgacacggaaatgttgaatactcatactcttcctttttcaatattattgaagcatttatcagggttattgtctcatgagcggatacatatttgaatgtatttagaaaaataaacaaataggggttccgcgcacatttccccgaaaagtgccac

Protocol for designing donor templates for HDR using the *y^wing2+^* dominant marker constructs

Overview:

Homology arms are cloned to span between the donor vector backbone and the yellow marker inserts to allow recombination to replace the locus of interest with the screenable marker. We will use the golden gate reaction to assemble the product:

Vector---upstream homology arm----GG..yellow dominant marker…CC-downstream homology arm---vector.

The GG and CC will produce new directional PAM sites allowing the original wild type sequence to be reinserted into the resultant fly strain without being cut by the new guide RNA + Cas9 combination. Internal sequences can thus be modified and reinserted to modify the locus of interest in any way desired.

For the reaction, directional restriction enzyme cuts will produce 4bp sticky ends with 5’ overhangs.

Put together in the following order by the golden gate reaction the result will be:

pBH…GACC…upstrmHMA…GGAT…yellow marker…TTCC…dwnstrmHMA…TATA…pBH

Where the color shaded sequences represent the overhangs between the respective pieces.

PROTOCOL:

1. define region (or gene) of interest (ROI - note that subsequent modifications are confined to the ROI)
   1. Use a suitable program to find Cas9 target sites flanking the ROI
   2. optional – it is helpful to build a sequence file as you design (see blank template below)
   3. recommended: verify sequence of actual fly strain to be injected at all sgRNA+PAM sites and all primer sites for PCR
   4. Choose enzyme for Golden Gate assembly (BbsI, BsaI or BsmBI) by examining the sequence ~1kb surrounding your region of interest for the presence of the enzyme. If all three enzymes are in the sequence, use Gibson of NEB HiFi assembly instead
      1. Choose upstream boundary of ROI by presence of suitable sgRNA+PAM sequences
         1. locate suitable PAM site flanking ROI upstream, note cut site (ie 3nts prior to NGG PAM relative to sense strand)
         2. Verify that nucleotides GG are not immediately downstream of cut site on sense orientation (this will interfere with cassette removal)
         3. Recommended: check and minimize predicted off-targets on same chromosome as ROI
         4. Recommended: check predicted efficiency of sgRNA >5.5 (<http://www.flyrnai.org/evaluateCrispr/>)
2. Predict novel sgRNA formed upon insertion (if cassette removal is desired. If only creating null mutant skip this step)
   - - 1. Note sequence of 21 nucleotides immediately upstream of cut site
       2. Add “GG” to end of 21 nucleotides immediately upstream of cut site = novel upstream sgRNA+PAM
       3. Recommended: check and minimize predicted off-targets on same chromosome as ROI
       4. Recommended: check predicted efficiency of sgRNA >5.5 (<http://www.flyrnai.org/evaluateCrispr/>)
       5. Optional: add up to 3 nucleotides between 21 nucleotide sequence and “GG” to increase specificity and/or predicted efficiency (these must be added to reverse primer for upstream homology arm – see below.
       6. If novel upstream sgRNA is not suitable, return to step i. 1 and choose another sgRNA+PAM sequence
       7. If novel upstream sgRNA is suitable (no precited off targets, efficiency >5.5), note the sequence of 20 nucleotides preceeding the NGG created (be sure to include optional 1-3 nucleotides if applicaple from step ii. 5.) and proceed to cloning upstream sgRNA from step i. and novel upstream sgRNA from step ii. (if cassette removal is desired) using appropriate protocol for sgRNA expression construct
     1. Clone upstream homology arm
        1. Design reverse primer
           1. Choose the annealing sequence for PCR= 18-30 nucleotides immediately upstream of the cutsite

Should end exactly at the cut site

Should have sufficient Tm for PCR

Should have little secondary structure and 40%-60% GC content, depending on Taq used

- - - - 1. Add the 1-3 nucleotides from step ii. 5. If applicable
        2. Add the overhang GGAT followed by the reverse orientation of the typeIIs restriction enzyme binding site chosen in step I. D
        3. Add 4-6 random nucleotids so the enzyme is not at the end of the sequence
        4. Reverse primer for upstream homology arm is **the reverse complement** of: a+b+c+d
      1. Design forward primer
         1. Add 4-6 random nucleotids so the enzyme is not at the end of the sequence
         2. Insert forward orientation of the typeIIs restriction enzyme binding site chosen in step I. D
         3. Add the overhang GACC
         4. Choose the annealing sequence for PCR= 18-30 nucleotides approximately 500-1000bp upstream of the cutsite

Should end exactly at the cut site

Should have sufficient Tm for PCR

Should have little secondary structure and 40%-60% GC content, depending on Taq used

- - - - 1. Forward primer for upstream homology arm is a+b+c+d
      1. PCR from wild-type genomic DNA from fly strain to be injected
    1. choose downstream boundary of ROI by presence of suitable sgRNA+PAM sequences
       1. locate suitable PAM site flanking ROI downstream, note cut site (ie 3nts prior to NGG PAM relative to sense strand)
       2. Verify that nucleotides CC are not immediately upstream of cut site on sense orientation (this will interfere with cassette removal)
       3. Recommended: check and minimize predicted off-targets on same chromosome as ROI (<http://tools.flycrispr.molbio.wisc.edu/targetFinder/>)
       4. Recommended: check predicted efficiency of sgRNA >5.5 (<http://www.flyrnai.org/evaluateCrispr/>)
    2. Predict novel sgRNA formed upon insertion (if cassette removal is desired. If only creating null mutant skip this step)
       1. Note sequence of 21 nucleotides immediately downstream of cut site
       2. Add “CC” to beginning of 21 nucleotides immediately downstream of cut site = novel downstream sgRNA+PAM
       3. Recommended: check and minimize predicted off-targets on same chromosome as ROI (<http://tools.flycrispr.molbio.wisc.edu/targetFinder/>)
       4. Recommended: check predicted efficiency of sgRNA >5.5 (<http://www.flyrnai.org/evaluateCrispr/>)
       5. Optional: add up to 3 nucleotides between 21 nucleotide sequence and “CC” to increase specificity and/or predicted efficiency (these must be added to reverse primer for upstream homology arm – see below.
       6. If novel downstream sgRNA is not suitable, return to step iii. 1 and choose another sgRNA+PAM sequence
       7. If novel downstream sgRNA is suitable (no predicted off targets, efficiency >5.5), note the sequence of 20 nucleotides preceding the NGG created (be sure to include optional 1-3 nucleotides if applicable from step ii. 5.) and proceed to cloning downstream sgRNA from step iii. and novel downstream sgRNA from step iv. (if cassette removal is desired) using appropriate protocol for sgRNA expression construct
    3. Clone downstream homology arm
       1. Design forward primer
          1. Add 4-6 random nucleotides so the enzyme is not at the end of the sequence
          2. Insert forward orientation of the typeIIs restriction enzyme binding site chosen in step I. D followed by the overhang TTCC
          3. Add the 1-3 nucleotides from step iv. 5. If applicable
          4. Choose the annealing sequence for PCR= 18-30 nucleotides immediately downstream of the cutsite

Should start exactly after the cut site

Should have sufficient Tm for PCR

Should have little secondary structure and 40%-60% GC content, depending on Taq used

- - - - 1. Forward primer for upstream homology arm is a+b+c+d
      1. Design reverse primer
         1. Choose the annealing sequence for PCR= 18-30 nucleotides approximately 500-1000 nucleotides downstream of the cutsite

Should start right after at the cut site

Should have sufficient Tm for PCR

Should have little secondary structure and 40%-60% GC content, depending on Taq used

- - - - 1. Add the 1-3 nucleotides from step iv. 5. If applicable
        2. Add the overhang TATA followed by the reverse orientation of the typeIIs restriction enzyme binding site chosen in step I. D
        3. Add 4-6 random nucleotides so the enzyme is not at the end of the sequence
        4. Reverse primer for downstream homology arm is **the reverse complement** of: a+b+c+d
      1. PCR from wild-type genomic DNA from fly strain to be injected
    1. Proceed to Golden Gate Assembly of HDR donor template plasmid

**Golden gate reaction using pBH as the destination vector**

Originally by Ben Housden – modified by David Li-Kroeger. 10 or (15ul rx)

1. PCR amplify upstream and downstream homology arms from genomic DNA prepared from the Cas9 line to be used for injection. Arms should be 500-1000bp each and should not contain cut sites for the restriction enzyme to be used for golden gate cloning.
2. Gel purify homology arms and normalize concentrations to 10ng/ul. (note: equimolar ratios of components is important)
3. Set up a golden gate reaction including the following:
   1. 15 (20ng) pBH-destination plasmid (2386bp)
   2. Equimolar amounts upstream homology arm
   3. Equimolar amounts downstream homology arm
   4. Equimolar amounts insert donor plasmid (ie y^wing2+^)
   5. 1 (1.5) ul buffer: cutsmart for BsaI; Buffer 2.1 for BbsI and for **BsmBI use T4 ligase buffer** (All buffers are NEB)
   6. 1 (1.5) ul 10mM ATP
   7. 0.5 (0.75) ul **high concentration** T4 ligase (NEB)
   8. 0.5 (0.75) ul restriction enzyme (BsaI, BbsI or BsmBI (NEB))
   9. water to 10 (15ul) total volume
4. Run golden gate program in a PCR machine:
   1. 37C – 3 mins
   2. 20C – 2 mins
   3. Goto a. 10-20X
   4. 37C – 5 mins
   5. Remove promptly and put on ice

**For BsmBI use the following conditions (remember to use buffer for T4 ligase)**

1. 1. 42C for 5mins
2. 2. 16C for 5mins
3. 3. Cycle 1-2 for 20times
4. 4. 60C for 10mins
5. 5. 80C for 10min
6. 6. 4C forever.
7. Transform in Stable2 cells and plate with kanamycin selection

Xform into stbl2 cells and Grow o/n at 30C

Select colonies and digest by restriction enzymes to give appropriate banding pattern to evaluate presence of both homology arms and insert

Sequence verify homology arms and flanking sequences of insert and backbone

Overhangs for typeIIs restriction enzymes

**BsaI**

UPSTREAM OVERHANG DOWNSTREAM OVERHANG

5’GGTCTCt 3’ 5’ NNNNcGAGACC 3’

3’CCAGAGaNNNN 5’ 3’ gCTCTGG 5’

**BbsI**

UPSTREAM OVERHANG DOWNSTREAM OVERHANG

5’GAAGACtt 3’ 5’ NNNNccGTCTTC 3’

3’CTTCTGaaNNNN 5’ 3’ ggCAGAAG 5’

**BsmBI**

UPSTREAM OVERHANG DOWNSTREAM OVERHANG

5’CGTCTCt 3’ 5’ NNNNcGAGACG 3’

3’GCAGAGaNNNN 5’ 3’ gCTCTGC 5’

pBH_y^wing2+^ blank template

insert sequence of Downstream homology arm here… (note that the ends may vary due to enzyme used - delete sequences in red to the appropriate TATA)

TATACGTCTCtTATAGAAGACttTATAttctagaGCCGTCCCGTCAAGTCAGCGTAATGCTCTGCCAGTGTTACAACCAATTAACCAATTCTGATTAGAAAAACTCATCGAGCATCAAATGAAACTGCAATTTATTCATATCAGGATTATCAATACCATATTTTTGAAAAAGCCGTTTCTGTAATGAAGGAGAAAACTCACCGAGGCAGTTCCATAGGATGGCAAGATCCTGGTATCGGTCTGCGATTCCGACTCGTCCAACATCAATACAACCTATTAATTTCCCCTCGTCAAAAATAAGGTTATCAAGTGAGAAATCACCATGAGTGACGACTGAATCCGGTGAGAATGGCAAAAGCTTATGCATTTCTTTCCAGACTTGTTCAACAGGCCAGCCATTACGCTCGTCATCAAAATCACTCGCATCAACCAAACCGTTATTCATTCGTGATTGCGCCTGAGCGAGtCGAAATACGCGATCGCTGTTAAAAGGACAATTACAAACAGGAATCGAATGCAACCGGCGCAGGAACACTGCCAGCGCATCAACAATATTTTCACCTGAgTCAGGATATTCTTCTAATACCTGGAATGCTGTTTTCCCGGGGATCGCAGTGGTGAGTAACCATGCATCATCAGGAGTACGGATAAAATGCTTGATGGTCGGAAGAGGCATAAATTCCGTCAGCCAGTTTAGTCTGACCATCTCATCTGTAACATCATTGGCAACGCTACCTTTGCCATGTTTCAGAAACAACTCTGGCGCATCGGGCTTCCCATACAATCGATAGATTGTCGCACCTGATTGCCCGACATTATCGCGAGCCCATTTATACCCATATAAATCAGCATCCATGTTGGAATTTAATCGCGGCCTGGAGCAAGACGTTTCCCGTTGAATATGGCTCATAACACCCCTTGTATTACTGTTTATGTAAGCAGACAGTTTTATTGTTCATGATGATATATTTTTATCTTGTGCAATGTAACATCAGAGATTTTGAGACATGAGCGTCAGACCCCGTAGAAAAGATCAAAGGATCTTCTTGAGATCCTTTTTTTCTGCGCGTAATCTGCTGCTTGCAAACAAAAAAACCACCGCTACCAGCGGTGGTTTGTTTGCCGGATCAAGAGCTACCAACTCTTTTTCCGAAGGTAACTGGCTTCAGCAGAGCGCAGATACCAAATACTGTTCTTCTAGTGTAGCCGTAGTTAGGCCACCACTTCAAGAACTCTGTAGCACCGCCTACATACCTCGCTCTGCTAATCCTGTTACCAGTGGCTGCTGCCAGTGGCGATAAGTCGTGTCTTACCGGGTTGGACTCAAGACGATAGTTACCGGATAAGGCGCAGCGGTCGGGCTGAACGGGGGGTTCGTGCACACAGCCCAGCTTGGAGCGAACGACCTACACCGAACTGAGATACCTACAGCGTGAGCtaTGAGAAAGCGCCACGCTTCCCGAAGGGAGAAAGGCGGACAGGTATCCGGTAAGCGGCAGGGTCGGAACAGGAGAGCGCACGAGGGAGCTTCCAGGGGGAAACGCCTGGTATCTTTATAGTCCTGTCGGGTTTCGCCACCTCTGACTTGAGCGTCGATTTTTGTGATGCTCGTCAGGGGGGCGGAGCCTATGGAAAAACGCCAGCAACGTCGActcgagGACCtGAGACCGACCtGAGACGGACCttGTCTTC

Insert sequence of the Upstream homology arm (note that the ends may vary due to enzyme used remove to the appropriate GACC)

GGATCTCTATTCAATCGGGACAGTGGAAATTGACTATTTTATTTATATTAATGAACTTATTTTTAATTTGGCTTAAGTTACTAAGGGGTACTAATAGTTTGAGCGCAGTGCATGTCATGGGGACATGTGCAATTGTGTGTAAGCGGGAAGTGATCGCGGCCTTCCGAATTTGGCCATGCCAAATAATCCCAGCTCGAAAGGAGGGGACCCGGCGGTCAGGGCCATGGACATTGAACTTGAAAAAAAAAAACACAAAAATATATAACACAAAACGGAAAATGCTGTGTACCGCTTATGTTAGAGAAGTTGAGCAACGGGTTTTTCGTTTTGCAGTCACGATGGATTTCCAAATTAGTGTAGGAGGGGGGAGGGGAGGGAGGGAGATAATGTCCAGGCTGCCATAAGTGGGGAATAAGGAAAATAAAACATGAAACACGGGTCGGGCAATGTCATGCGGTATTCGGCTTTGCTTTCCGCCCAAGTTGAAGTGATCCTGTGTGTAAATAATGTCGAATGTTGCCGGTCGGTTGCATAAGCGTTAGTcaattatggccaaagagatctgatttgtggaGTCGAGAAAAAAACCTTCATATAAAACGCGGCCGACATATTATGGCCACCAGTCGTTACCGCGCCACGGTCCACAGAAGAGGATTAAAAAAATATCACACAGCCGAAGGCTAGAGAAGAACCCCCTATAGCTGAACATATATAAACAAATATATTTTTTTTTATTGCCAACACACTTTGGCTTAAGTGTTAAGAGTGATTGTCAGCTTAGAGCTAAGTGCAATGTTCCAGGACAAAGGGTGGATCCTTGTGACCCTGATCACCTTGGTGACGCCGTCTTGGGCTGCTTACAAACTTCAGGAGCGATATAGTTGGAGCCAGCTGGACTTTGCTTTCCCGAATACCCGACTAAAGGACCAAGCTCTGGCTAGTGGAGATTATATTCCGCAAAATGCTCTACCTGTTGGAGTCGAACACTTTGGCAATCGGTTATTCGTCACTGTTCCCCGCTGGCGTGATGGGATTCCGGCCACTCTGACCTATATAAACATGGACCGCAGTTTGACGGGTTCACCGGAGCTAATTCCGTATCCAGATTGGCGCTCAAATACAGCTGGAGATTGCGCCAACAGTATTACCACTGCCTACCGCATTAAAGTGGATGAGTGTGGTCGGCTGTGGGTTTTGGACACTGGAACCGTGGGCATCGGCAATACCACCACTAATCCGTGCTCCTATGCGGTAAATGTCTTTGACTTGACCACGGATACGCGAATTCGGAGATACGAGCTACCTGGCGTGGACACAAATCCAAATACTTTCATAGCTAACATTGCCGTGGATATAGGCAAAAATTGCGATGATGCATATGCCTATTTTGCCGATGAATTGGGATACGGCTTGATTGCTTACTCCTGGGAACTGAACAAGTCCTGGAGATTCTCGGCACATTCGTATTTTTTCCCCGATCCATTGAGGGGCGATTTCAATGTCGCTGGTATTAACTTCCAATGGGGCGAGGAGGGTATATTTGGTATGTCCCTTTCGCCCATTCGATCGGATGGTTATCGTACCCTGTACTTTAGTCCGTTAGCAAGTCATCGACAATTTGCCGTATCCACGAGGATTTTGAGGGATGAAACCAGGACGGAAGATAGCTATCATGACTTTGTTGCCTTAGATGAACGGGGTCCAAACTCCCATACCACTTCACGTGTGATGAGCGATGATGGAATTGAGCTGTTCAATTTAATAGATCAAAATGCAGTGGGTTGCTGGCACTCATCAATGCCGTACTCACCGCAATTTCATGGCATTGTGGATCGCGATGACGTTGGCTTAGTTTTTCCGGCCGATGTGAAAATTGATGAGAACAAAAACGTTTGGGTTCTATCCGATAGGATGCCCGTTTTCTTGCTGTCTGACTTGGATTATTCAGATACTAATTTCCGAATTTACACGGCTCCCTTGGCCACTTTAATTGAGAATACTGTGTGTGATTTGAGGAATAACGCCTATGGGCCGCCAAATACCGTTTCAATACCAAAACAAGCCGTTTTGCCAATGGGTCCACCGTTATATACGAAACAATATCGTCCTGTCTTGCCACAGAAACCTCAGACCAGCTGGGCTTCCTCGCCGCCTCCTCCAAGTCGCACTTATTTGCCCGCCAATTCAGGCAATGTAGTCTCCAGTATTAGTGTCTCTACAAATTCTGTGGGTCCTGCAGGAGTGGAGGTGCCAAAGGCCTATATTTTCAACCAGCACAACGGCATAAATTACGAGACAAGTGGTCCCCATCTATTTCCCACCCATCAACCCGCCCAACCGGGTGGCCAGGATGGTGGGTTAAAAACTTATGTGAATGCCCGCCAATCTGGGTGGTGGCATCATCAGCATCAAGGTTAACATAATCCTACACACGGTACTTGGGTATATTCTCACACACTCGATTGATGTAAAGAATATTTAAAGACAACAACATAGGGCAACAGCGGTTAAAAAAACCACATGACGTATGAGCAAGTGGCAAATCAATACTTTATCTAGTTATGTTAAGCAAAAAATAACAATAAATCAACTTTTTTTTGAAGGTTAAGAGTTTACGCAATTTTCTTGAGCGGAAAAAGCGGAAAAAATGTAAGTATGCATAAATTCTAAATATATCAACAACTGTACATTTTCTGGAGTACTACTACCAGGCAAGAAAGTAGGTTGATAAAGCTATGCACAAGATCTTGTTTGGGTGCAGGGAAAGTTCAACTTAATCGCTCAATTTGAGATCGCCTGGTCGCTTGAGATTCGACTGTAATTGAAATTTTTGCTTTTGATCGGAGCCAGACTTCAGACGGGGCAAACAAAAAGACTTTGTTGGTGGTAGGGTAGGATCCGTTGACCTGCAGGTTCC

oligos

sequencing from pBH forward GCCACCTCTGACTTGAGCGTCG

sequencing from pBH reverse GAGTTTTCTCCTTCATTACAGAAACGGC

sequencing from ywing2+ upstream HMA reverse GCACTGCGCTCAAACTATTAGTACCCC

sequencing from ywing2+ downstream HMA forward CGGAGCCAGACTTCAGACGGG
